# Epigenetic Control of Translation Checkpoint and Tumor Progression via RUVBL1‐EEF1A1 Axis

**DOI:** 10.1002/advs.202206584

**Published:** 2023-04-19

**Authors:** Mingli Li, Lu Yang, Anthony K. N. Chan, Sheela Pangeni Pokharel, Qiao Liu, Nicole Mattson, Xiaobao Xu, Wen‐Han Chang, Kazuya Miyashita, Priyanka Singh, Leisi Zhang, Maggie Li, Jun Wu, Jinhui Wang, Bryan Chen, Lai N. Chan, Jaewoong Lee, Xu Hannah Zhang, Steven T. Rosen, Markus Müschen, Jun Qi, Jianjun Chen, Kevin Hiom, Alexander J. R. Bishop, Chun‐Wei Chen

**Affiliations:** ^1^ Department of Systems Biology Beckman Research Institute City of Hope Comprehensive Cancer Center Duarte CA 91010 USA; ^2^ Division of Epigenetic and Transcriptional Engineering Beckman Research Institute City of Hope Comprehensive Cancer Center Duarte CA 91010 USA; ^3^ City of Hope Comprehensive Cancer Center Duarte CA 91010 USA; ^4^ Center of Molecular and Cellular Oncology Yale Cancer Center Yale School of Medicine New Haven CT 06510 USA; ^5^ Department of Cancer Biology Lerner Research Institute Cleveland Clinic Cleveland OH 44195 USA; ^6^ School of Biosystems and Biomedical Sciences College of Health Science Korea University Seoul 02841 South Korea; ^7^ Interdisciplinary Program in Precision Public Health Korea University Seoul 02841 South Korea; ^8^ Department of Cancer Biology Dana‐Farber Cancer Institute Harvard Medical School Boston MA 02215 USA; ^9^ Division of Cellular Medicine School of Medicine University of Dundee Nethergate Dundee DD1 4HN UK; ^10^ Department of Cellular Systems and Anatomy University of Texas Health Science Center at San Antonio San Antonio TX 78229 USA; ^11^ Greehey Children's Cancer Research Institute University of Texas Health Science Center at San Antonio San Antonio TX 78229 USA

**Keywords:** EEF1A1, epigenetic, Ewing sarcoma, KAT5, MYC, RUVBL1

## Abstract

Epigenetic dysregulation is reported in multiple cancers including Ewing sarcoma (EwS). However, the epigenetic networks underlying the maintenance of oncogenic signaling and therapeutic response remain unclear. Using a series of epigenetics‐ and complex‐focused CRISPR screens, RUVBL1, the ATPase component of NuA4 histone acetyltransferase complex, is identified to be essential for EwS tumor progression. Suppression of RUVBL1 leads to attenuated tumor growth, loss of histone H4 acetylation, and ablated MYC signaling. Mechanistically, RUVBL1 controls MYC chromatin binding and modulates the MYC‐driven EEF1A1 expression and thus protein synthesis. High‐density CRISPR gene body scan pinpoints the critical MYC interacting residue in RUVBL1. Finally, this study reveals the synergism between RUVBL1 suppression and pharmacological inhibition of MYC in EwS xenografts and patient‐derived samples. These results indicate that the dynamic interplay between chromatin remodelers, oncogenic transcription factors, and protein translation machinery can provide novel opportunities for combination cancer therapy.

## Graphical abstract

Dysregulation of MYC family proto‐oncogene is a hallmark of tumorigenesis in various cancers. This study demonstrates that RUVBL1 is a critical modulator of MYC‐mediated EEF1A1 expression and protein synthesis. RUVBL1 serves as a master regulator of oncogenic MYC signaling via controlling MYC chromatin binding. Targeting RUVBL1 synergizes with the pharmacological targeting of MYC, pointing toward a more effective combinatorial therapy.

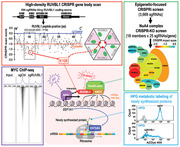

## 1 Introduction

Ewing sarcoma (EwS), one of the most common bone and soft tissue malignancies in children and young adults,^[^ ^1^ ^]^ is driven by a chromosomal translocation that fuses the N‐terminus of Ewing Sarcoma Breakpoint Region 1 (EWSR1) to the C‐terminus of the ETS family of transcription factors (e.g., FLI1, ERG, etc.), leading to the expression of EwS‐fusion oncoproteins.^[^ ^2^ ^]^ These fusion oncoproteins bind to the GGAA motif‐containing loci or microsatellites^[^ ^3^ ^]^ on chromatin by acting as aberrant transcription factors, resulting in gene expression changes including the activation of oncogenic MYC, repression of tumor suppressors IGFBP3, etc.^[^ ^4^ ^]^ Furthermore, recent reports revealed EwS is among the most “mutation cold” tumors,^[^ ^5^ ^]^ arguing a limited number of genetic candidates to serve as therapeutic targets. On the other hand, EwS‐fusions have been reported to recruit chromatin remodeling/modifying machinery such as the SWI/SNF complex.^[^ ^6^ ^]^ These observations suggest nongenetic mechanisms underlying the EwS disease progression that the transformed EwS cells may adopt a novel epigenetic state,^[^ ^7^ ^]^ thereby, bypasses the normal mesenchymal maturation. Hence, targeting the indispensable epigenetic circuitry in EwS can provide alternative and more effective therapeutic options.

In this study, we conducted an unbiased epigenetics‐focused CRISPR interference (CRISPRi)^[^ ^8^ ^]^ library screen and identified the requirement of the Nucleosome acetyltransferase of histone H4 (NuA4) complex^[^ ^9^ ^]^ in EwS maintenance. NuA4 is a multiprotein complex involved in transcriptional activation and DNA damage repair by acetylation of nucleosomal histones.^[^ ^9^, ^10^ ^]^ Using histone proteomics (mass spec), epigenetics (ChIP‐seq), and transcriptomics (RNA‐seq) profiling, our study collectively revealed that RuvB like AAA ATPase 1 (RUVBL1; also known as PONTIN or TIP49),^[^ ^11^ ^]^ the ATPase component of NuA4 complex, is essential for the maintenance of KAT5 (also known as TIP60 or yeast Esa1)‐mediated histone H4 acetylation^[^ ^10^, ^12^ ^]^ and MYC chromatin binding. Dysregulation of MYC family proto‐oncogene has been reported in various cancers.^[^ ^13^ ^]^ For example, EWSR1‐FLI1 fusion oncoprotein can drive an excessive expression of MYC^[^ ^4a^ ^]^ to support EwS proliferation.^[^ ^14^ ^]^ Furthermore, the capacity of RUVBL1 and other NuA4 members to interact with MYC and modulate the MYC transcription activity has been documented in multiple organisms.^[^ ^15^ ^]^ The major downstream of the oncogenic MYC signaling supports cell‐cycle progression, ribosome biogenesis, cell survival, and energy metabolism; thus, inhibition of MYC via targeting NuA4 complex represents an attractive opportunity for cancer therapy.

Our study also utilized a high‐density CRISPR tilling screen approach^[^ ^16^ ^]^ and identified that the lysine 108 (K108) of RUVBL1 as essential for the interaction between RUVBL1 and MYC. This novel interaction site is crucial to the RUVBL1‐MYC feed‐forward network and the expression of Eukaryotic Translation Elongation Factor 1 Alpha 1 (EEF1A1). EEF1A1 is one of the most abundant proteins found in eukaryotic proteomes and a critical nonribosomal component of the translational machinery that supports protein translation elongation.^[^ ^17^ ^]^ EEF1A1 binds guanine nucleotides and delivers the aminoacyl‐tRNAs to the ribosomal A‐site in a GTPase‐dependent manner.^[^ ^18^ ^]^ Furthermore, the upregulation of protein translational output by modulating EEF1A1 activity has been reported to promote tumorigenesis.^[^ ^19^ ^]^ Our study identified a novel epigenetic regulation that controls the overall protein translation throughput via a RUVBL1‐MYC‐EEF1A1 axis. These notions also led us to demonstrate the potential of targeting RUVBL1 as a novel therapeutic strategy against EwS.

## 2 Results

### 2.1 Serial CRISPR Screens Identify RUVBL1 as a Novel Vulnerability in EwS

**Figure 1:**
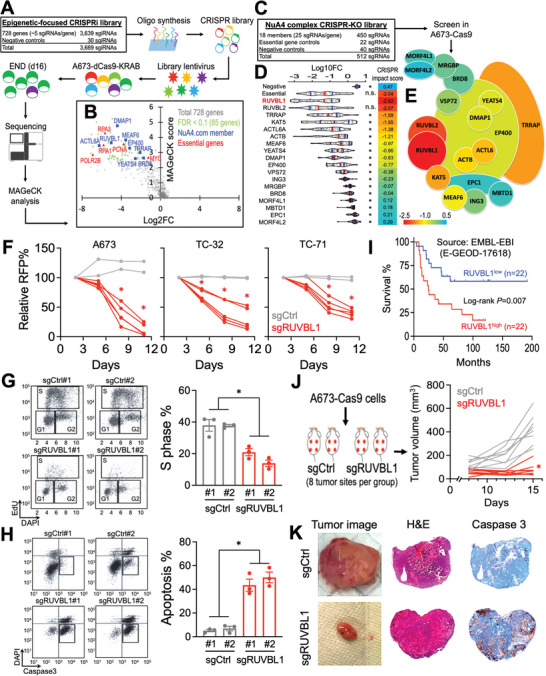
Serial CRISPR screens identify the essential role of RUVBL1 in EwS. A) Schematic outline of an epigenetic‐focused CRISPRi screen in A673‐dCas9‐KRAB cells. B) Volcano plot depicts the log2 fold change of sgRNA abundance during 16 d of screen culture (*x*‐axis; log2FC) and the significance (*y*‐axis; MAGeCK score) of each gene in the epigenetics CRISPRi screen (*n* = 3 replicates). C) Library design of the NuA4 complex CRISPR screen in A673‐Cas9 cells. D) Violin plots indicate the median (red lines), first and third quartiles (blue lines), and the log10 fold change of individual sgRNA (dots) during 16 d of NuA4 complex CRISPR screen culture (*n* = 3 replicates). E) Heatmap showing the CRISPR impact scores (Log10FC of the first quartile out of 25 sgRNAs per gene) of each member in the NuA4 complex CRISPR screen. F) Growth competition assay of Cas9‐expressing A673, TC‐32, and TC‐71 EwS cells transduced with RFP‐labeled negative control sgRNAs (gray lines; *n* = 2 independent sgCtrl sequences) and sgRNAs targeting RUVBL1 (red lines; *n* = 5 independent sgRUVBL1 sequences). G) Cell cycle monitored by EdU incorporation, and H) cellular apoptosis detected by active caspase 3^+^/DAPI^−^ in A673‐Cas9 cells transduced with sgCtrl and sgRUVBL1 for 7 d (*n* = 3 for each group). I) Survival curves of patients with EwS family of tumors expressing high versus low RUVBL1 (22 patients for each group). J) Profile plot of EwS xenograft tumor volume in mice inoculated with sgCtrl and sgRUVBL1 transduced A673‐Cas9 cells (*n* = 8 tumor sites per group). K) Tumor image (left), hematoxylin and eosin stain (middle), and cleaved caspase 3 stain (right; brown) of sgCtrl and sgRUVBL1 transduced A673‐Cas9 xenograft tumor. Data are represented as mean ± SEM. *P < 0.01 compared to D) RUVBL1 and F–H,J) sgCtrl by two‐sided Student's *t*‐test. Source data are available for this figure: SourceData F1 A, B, and D.

To characterize critical epigenetic mechanisms supporting the transformed EwS cells, we developed a custom CRISPRi library (total of 3669 sgiRNAs) targeting the transcription start site (TSS) of 728 epigenetic‐related genes in the human genome (**Figure** **1**A; Figure S1A,B, Supporting Information). We then delivered this library into the A673 cells (a well‐established EWSR1‐FLI1 fusion EwS cell model that is amenable to CRISPR genetic screen and in vivo xenograft)^[^ ^5^, ^20^ ^]^ stably expressing an enzymatic‐inactivated Cas9 fusion with the transcription repressor KRAB (i.e., A673‐dCas9‐KRAB cells; Figure S2A, Supporting Information) using lentiviral transduction, and compared the change of frequency of each integrated sgRNA construct in these cells between day 0 and day 16 using high‐throughput sequencing followed by MAGeCK algorithm^[^ ^21^ ^]^ (Figure 1B; SourceData 1 and 2). In addition to the genes commonly essential to cancer cells (red dots), we observed a cluster of eight genes belonging to the mammalian NuA4 histone acetyltransferase complex (blue dots)^[^ ^9^ ^]^ within the 85 candidate genes in the screen (FDR < 0.1), marking this complex as the top essential chromatin effectors in EwS cells.

NuA4 is a multi‐subunit complex that consists of 18 protein members.^[^ ^9a^ ^]^ To pinpoint the critical effectors within the NuA4 complex, we developed another CRISPR library with 25 sgRNAs targeting the coding regions of each NuA4 member gene for a CRISPR depletion screen in the A673‐Cas9 cells (Figure 1C; Figures S1C and S2B, Supporting Information). This orthogonal CRISPR screen using distinct sgRNA sequences and gene suppression mechanisms from the primary CRISPRi screen (i.e., five sgiRNAs targeting each gene's TSS) revealed RUVBL1 as the most critical member of NuA4 in A673 cells (Figure 1D,E; SourceData 3). CRISPR depletion of RUVBL1 in A673, TC‐32, and TC‐71 EwS cells resulted in suppression of cell proliferation (Figure 1F; Figure S3 and Table S1, Supporting Information), which is associated with arrested cell cycle and pronounced apoptosis (Figure 1G,H). Clinically, we observed an association of high RUVBL1 expression level with poor survival prognosis in patients with EwS family of tumors (Figure 1I; Figure S4, Supporting Information; including EwS, Askin tumors, and primitive neuroectodermal tumors [PNET]). Finally, CRISPR depletion of RUVBL1 significantly retarded the EwS tumor progression (Figure 1J; sgCtrl = 423.5 ± 44.5 mm^3^; sgRUVBL1 = 77.9 ± 18.1 mm^3^; data represent day 15 mean tumor volume ± SEM) with a drastic induction of cleaved caspase 3 staining (Figure 1K; an apoptotic marker) in the A673 xenograft model, indicating the indispensable role of RUVBL1 in EwS maintenance.

### 2.2 Transcription and Chromatin Profiling Revealed a Feed‐Forward Network between RUVBL1 and MYC

**Figure 2:**
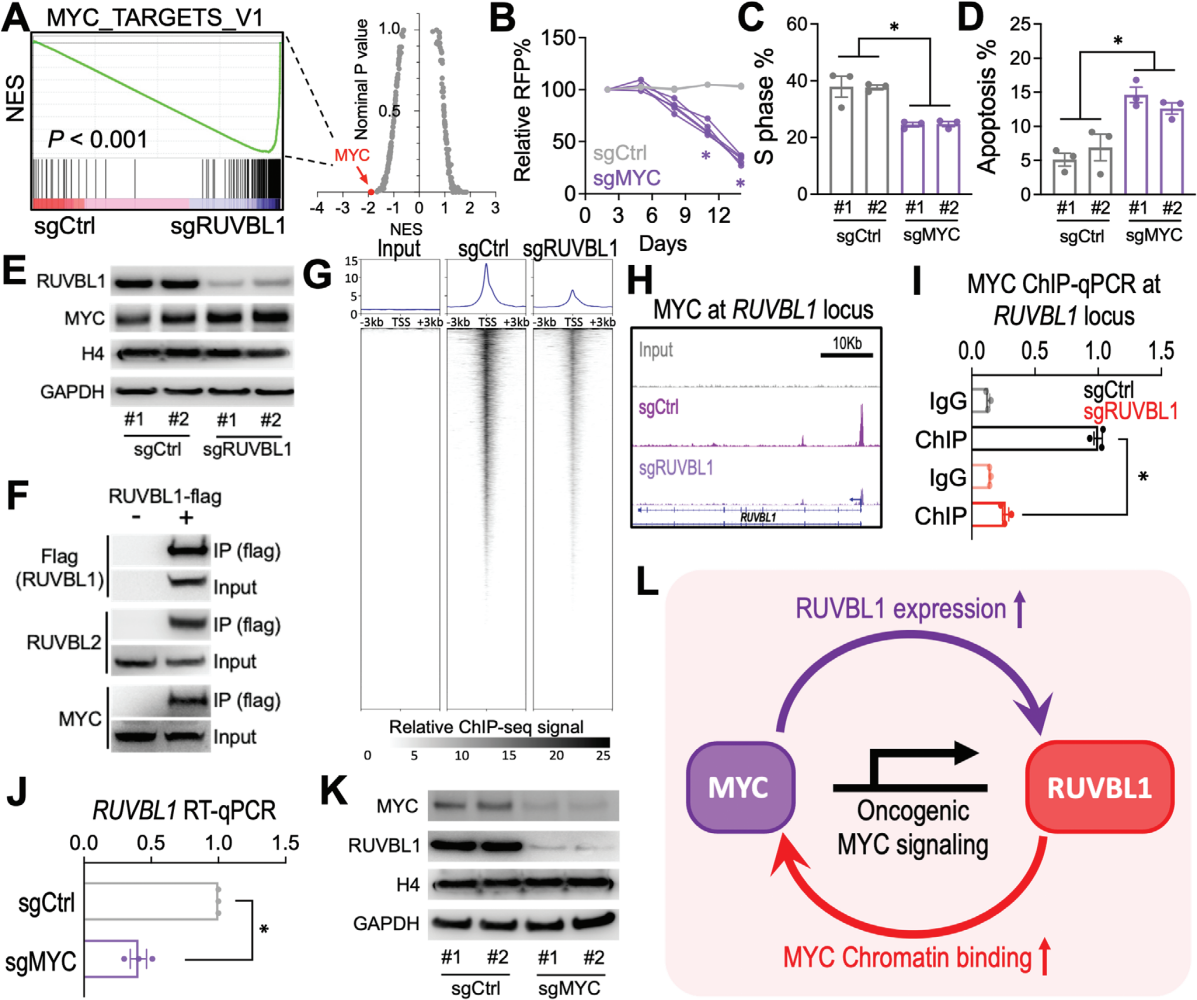
RUVBL1 controls MYC chromatin localization and transactivation activity in EwS. A) RNAseq and GSEA analyses showing changes in expression of the MYC upregulated target gene set in sgCtrl and sgRUVBL1 transduced (day 5) A673‐Cas9 cells (two independent sgRNA sequences per group). (Right) Each dot indicates one gene set from the GSEA Molecular Signature Database (MSigDB; total 238 gene sets from the Hallmark and Oncogenic Signature [C6] collections). NES: Normalized enrichment score. B) Growth competition assay of sgCtrl (gray lines; *n* = 2 independent sgRNA sequences) and sgMYC (purple lines; *n* = 5 independent sgRNA sequences) in A673‐Cas9 cells. C) Cell cycle monitored by EdU incorporation, and D) cellular apoptosis detected by active caspase 3^+^/DAPI^−^ in A673‐Cas9 cells transduced with sgCtrl and sgMYC (*n* = 3 for each group). E) Western blot of RUVBL1, MYC, histone H4, and GAPDH in A673‐Cas9 cells transduced with sgCtrl and sgRUVBL1 (two independent sgRNA sequences per group). F) Co‐IP of RUVBL1 (flag‐tagged) with RUVBL2 and MYC in HEK293 cells. G) Meta plots (top) and heatmaps (bottom) showing ChIP‐seq signal of MYC at TSSs ± 3 kb regions for all genes in A673‐Cas9 cells transduced with sgCtrl and sgRUVBL1. H) Profiles of MYC ChIP‐seq and (I) ChIP‐qPCR at *RUVBL1* locus in A673‐Cas9 cells transduced with sgCtrl and sgRUVBL1 (*n* = 3 for each group). J) RT‐qPCR of *RUVBL1* mRNA in A673‐Cas9 cells transduced with sgCtrl and sgMYC (*n* = 3 for each group). K) Western blot of MYC, RUVBL1, and GAPDH in A673‐Cas9 cells transduced with sgMYC (two independent sgRNA sequences per group). L) Model of a feed‐forward network between RUVBL1 and MYC in EwS. Data are represented as mean ± SEM. *P < 0.01 compared to sgCtrl by two‐sided Student's *t*‐test.

To elucidate the transcriptomic impact induced by depletion of RUVBL1, we performed RNA‐seq and gene set enrichment analysis (GSEA) on A673‐Cas9 cells transduced with sgCtrl versus sgRUVBL1. We found that, compared to the control, the “MYC‐target gene signature” is amongst the most depleted gene sets upon sgRUVBL1 transduction (**Figure** **2**A). Similar to sgRUVBL1, depletion of MYC by CRISPR significantly suppressed the proliferation and survival of the EwS cells (Figure 2B–D), indicating an essential role of the oncogenic MYC signaling in EwS maintenance. Intriguingly, the MYC expression was not inhibited by the depletion of RUVBL1 (Figure 2E). Nonetheless, we observed the participation of MYC in the RUVBL1‐containing complex (Figure 2F), arguing the involvement of RUVBL1 in MYC's oncogenic function through protein–protein interaction.^[^ ^15a^ ^]^

MYC is a transcription activator that binds to chromatin and mediates the expression of its target genes.^[^ ^22^ ^]^ To examine the impact of RUVBL1 on MYC's chromatin targeting, we captured the MYC‐associated chromatin in sgCtrl and sgRUVBL1 transduced A673 EwS cells for high‐throughput sequencing (ChIP‐seq; Figure S5, Supporting Information). Our results revealed a remarked reduction of MYC occupancy at its chromatin targets in the sgRUVBL1 cells (Figure 2G), highlighting the capacity of RUVBL1 to control MYC's function via mediating chromatin targeting. It is important to note that while the interaction between RUVBL1 and MYC has been previously reported,^[^ ^15a^ ^]^ the role of RUVBL1 as a master regulator of MYC's chromatin binding (Figure S6, Supporting Information) was not previously noted. Furthermore, we observed that the *RUVBL1* gene locus is an MYC‐bound target, and sgRUVBL1 eliminated MYC binding at *RUVBL1*’s TSS (Figure 2H,I). CRISPR depletion of MYC reduced the expression of RUVBL1 at both mRNA and protein levels (Figure 2J,K). These results implicate a feed‐forward relationship between RUVBL1 and MYC in maintaining the oncogenic program (Figure 2L).

### 2.3 RUVBL1 Controls MYC‐Driven EEF1A1 Expression and Protein Synthesis

**Figure 3:**
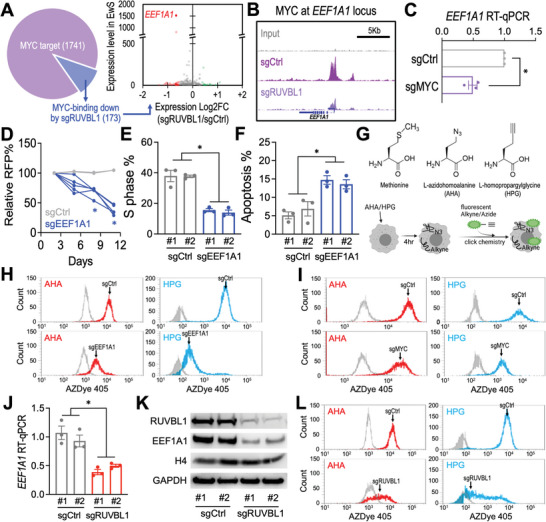
RUVBL1 controls protein synthesis via mediating EEF1A1 expression. A) RNAseq evaluation of the expression level in EwS (*y*‐axis) and the log2 fold change of expression induced by sgRUVBL1 (*x*‐axis) of the RUVBL1‐regulated MYC targets (173 genes). The depleted (red) and enriched (green) genes are highlighted. B) Profiles of MYC ChIP‐seq at *EEF1A1* locus in A673‐Cas9 cells transduced with sgCtrl and sgRUVBL1. C) RT‐qPCR of *EEF1A1* mRNA in A673‐Cas9 cells transduced with sgCtrl and sgMYC (*n* = 3 for each group). D) Growth competition assay of sgCtrl (gray lines; *n* = 2 independent sgRNA sequences) and sgEEF1A1 (blue lines; *n* = 5 independent sgRNA sequences) in A673‐Cas9 cells. E) Cell cycle monitored by EdU incorporation, and F) cellular apoptosis detected by active caspase 3^+^/DAPI^−^ in A673‐Cas9 cells transduced with sgCtrl and sgEEF1A1 (*n* = 3 for each group). G) Schematic outline of metabolic labeling of the newly synthesized proteins using AHA/HPG incorporation. Flow cytometric profiles of AHA (red) and HPG (cyan) labeled compared to the nonlabeled (gray) cells in A673‐Cas9 cultures transduced with H) sgCtrl versus sgEEF1A1, I) sgCtrl versus sgMYC, and L) sgCtrl versus sgRUVBL1. J) RT‐qPCR of *EEF1A1* mRNA in A673‐Cas9 cells transduced with sgCtrl and sgRUVBL1 (two independent sgRNA sequences; *n* = 3 for each group). K) Western blot of RUVBL1, EEF1A1, histone H4, and GAPDH in A673‐Cas9 cells transduced with sgCtrl and sgRUVBL1 (two independent sgRNA sequences per group). Data are represented as mean ± SEM. *P < 0.01 compared to sgCtrl by two‐sided Student's *t*‐test.

To identify the critical oncogenic effectors regulated by the RUVBL1/MYC feed‐forward network, we first identified 1741 MYC target genes (i.e., more than tenfold enrichment of MYC ChIP‐seq signal over input at TSS ± 1 kb) and found that 173 of these genes showed more than 40% reduction in MYC binding signal upon RUVBL1 depletion (**Figure** **3**A; Figure S6B, Supporting Information). Out of these RUVBL1/MYC‐coregulated genes, we identified a significant depletion of a highly expressed gene *EEF1A1* in sgRUVBL1 transduced cells. The *EEF1A1* locus showed a reduced MYC binding signal upon RUVBL1 deletion (Figure 3B), and sgMYC reduced the level of *EEF1A1* transcript (Figure 3C), suggesting an MYC‐EEF1A1 axis downstream of RUVBL1.

EEF1A1 is an associated component of the ribosomal complex that supports the protein translation elongation via delivering the aminoacyl‐tRNAs to the ribosomal A site.^[^ ^17^, ^18^, ^19^ ^]^ CRISPR depletion of EEF1A1 impaired the proliferation and survival of the EwS cells (Figure 3D–F), phenocopying the effect of sgRUVBL1 (Figure 1F–H). Furthermore, by monitoring the incorporation of l‐azidohomoalanine and l‐homopropargylglycine (AHA and HPG; both are analogs of methionine) into the newly synthesized proteins (Figure 3G), we observed a drastic loss of protein synthesis in the sgEEF1A1 transduced cells (Figure 3H). Importantly, CRISPR depletion of MYC phenocopied the reduced AHA/HPG incorporation observed in EEF1A1 depleted cells (Figure 3I). Similarly, the reduced EEF1A1 expression and impaired protein synthesis capacity were also observed in the sgRUVBL1 targeted cells (Figure 3J–L). Taken together, our results nominated EEF1A1 as the protein translation checkpoint underlying the RUVBL1/MYC transcriptional network to control protein synthesis in EwS cells.

### 2.4 RUVBL1 Recruits Lysine Acetyltransferase 5 (KAT5) and Modulates Histone H4 Acetylation

**Figure 4:**
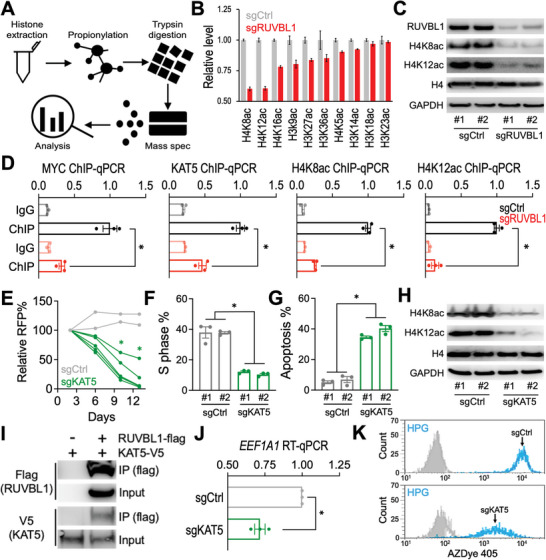
RUVBL1 modulates histone H4 acetylation via KAT5. A) Schematic outline of histone modification mass spectrometry and B) levels of histone H3 and H4 acetylation detected in sgCtrl and sgRUVBL1 transduced A673‐Cas9 cells (*n* = 3 mass spec measurements per sample). C) Western blot of RUVBL1, H4K8ac, H4K12ac, histone H4, and GAPDH in A673‐Cas9 cells transduced with sgCtrl versus sgRUVBL1 (two independent sgRNA sequences per group). D) ChIP‐qPCR of MYC, KAT5, H4K8ac, and H4K12ac at *EEF1A1* locus in A673‐Cas9 cells transduced with sgCtrl and sgRUVBL1 (*n* = 3 for each group). E) Growth competition assay of sgCtrl (gray lines; *n* = 2 independent sgRNA sequences) and sgKAT5 (green lines; *n* = 5 independent sgRNA sequences) in A673‐Cas9 cells. F) Cell cycle monitored by EdU incorporation, and G) cellular apoptosis detected by active caspase 3^+^/DAPI^−^ in A673‐Cas9 cells transduced with sgCtrl and sgKAT5 (*n* = 3 for each group). H) Western blot of RUVBL1, H4K8ac, H4K12ac, histone H4, and GAPDH in A673‐Cas9 cells transduced with sgCtrl versus sgKAT5 (two independent sgRNA sequences per group). I) Co‐IP of RUVBL1 (flag‐tagged) with KAT5 (V5‐tagged) in HEK293 cells. J) RT‐qPCR of *EEF1A1* mRNA in A673‐Cas9 cells transduced with sgCtrl and sgKAT5 (*n* = 3 for each group). K) Flow cytometric profiles of HPG labeled (cyan) compared to the nonlabeled (gray) cells in A673‐Cas9 cultures transduced with sgCtrl versus sgKAT5. Data are represented as mean ± SEM. *P < 0.01 compared to sgCtrl by two‐sided Student's *t*‐test. Source data are available for this figure: SourceData F4 B.

As a histone acetyltransferase complex, NuA4 is responsible for the acetylation of histone H4 N‐terminal tails,^[^ ^23^ ^]^ which are chromatin modifications highly associated with transcription initiation and gene expression.^[^ ^24^ ^]^ To investigate the epigenetic role of RUVBL1 in EwS, we quantified the major acetylation positions on histone H3/H4 using mass spectrometry (**Figure** **4**A) and observed a pronounced loss of acetylation at H4K8 and H4K12 upon sgRUVBL1 transduction (Figure 4B; SourceData 4). Similar results were also observed when we utilized the site‐specific histone acetylation antibodies and immunoblotting (Figure 4C; Figure S7A, Supporting Information).

While RUVBL1 does not have a predicted acetyltransferase activity, we turned our attention to the catalytic components of the NuA4 complex called KAT5^[^ ^9b^ ^]^ (Figure 1D,E; the fourth hit in the NuA4 complex screen), which is also a known co‐factor of MYC.^[^ ^12a^ ^]^ Depletion of RUVBL1 decreased the localization of MYC at the *EEF1A1* locus, which is concomitant with the reduced levels of KAT5, H4K8ac, and H4K12ac (Figure 4D). Furthermore, CRISPR depletion of KAT5 resembled the loss of cell fitness (Figure 4E–G) and the reduced H4K8ac/H4K12ac (Figure 4H; Figure S7B, Supporting Information) observed in sgRUVBL1 transduced EwS cells (Figures 1F–H, and 4C). Co‐immunoprecipitation (co‐IP) of RUVBL1 detected the participation of KAT5 in the RUVBL1‐containing complex (Figure 4I).^[^ ^9a^ ^]^ Finally, sgKAT5 significantly reduced the level of *EEF1A1* transcript (Figure 4J) and attenuated the rate of protein synthesis (Figure 4K), phenocopying the effect exerted by sgRUVBL1 (Figure 3J,L).

### 2.5 High‐Density CRISPR Gene Body Scan Identifies a Novel MYC‐Interacting site in RUVBL1

**Figure 5:**
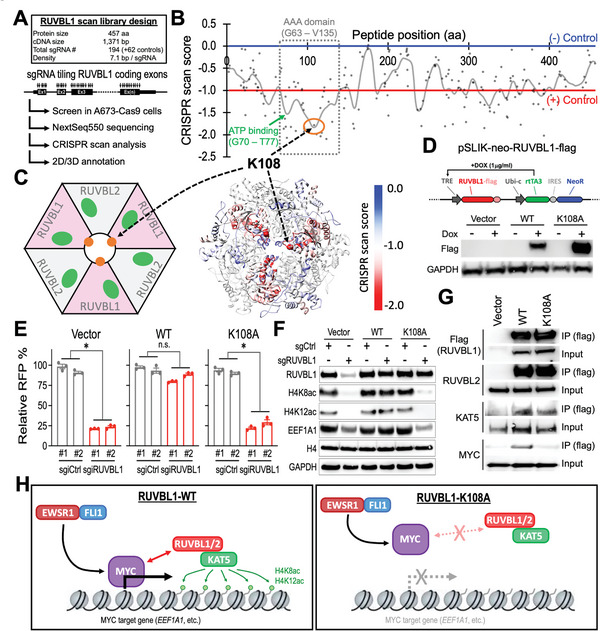
Lysine 108 in RUVBL1 is required for the interaction between RUVBL1 and MYC. A) Schematic outline of RUVBL1 high‐density CRISPR gene body scan in A673‐Cas9 cells. B) 2D annotation of RUVBL1 CRISPR scan. The gray line indicates the smoothened model of the CRISPR scan score derived from 194 sgRNAs (dots) targeting the coding exons of RUVBL1 (*n* = 3 replicates). The median CRISPR scan scores of the positive control (red line; defined as −1.0) and negative control (blue line; defined as 0.0) sgRNAs are highlighted. C) 3D annotation RUVBL1 CRISPR scan score relative to a cryo‐EM structural model of a hexamer consists of three RUVBL1 and three RUVBL2 proteins (PDB ID: 5OAF). D) Western blot showing doxycycline (DOX)‐induced expression of flag‐tagged WT‐ and K108A‐RUVBL1 in A673‐dCas9‐KRAB cells. E) Effect of WT‐ and K108A‐RUVBL1 expression on the growth competition assay of A673‐dCas9‐KRAB cells transduced with sgiCtrl and sgiRUVBL1 (*n* = 3 for each group). F) Western blot of RUVBL1, H4K8ac, H4K12ac, EEF1A1, histone H4, and GAPDH in WT‐ and K108A‐RUVBL1 expressing A673‐dCas9‐KRAB cells transduced with sgiCtrl and sgiRUVBL1. G) Co‐IP of WT‐ and K108A‐RUVBL1 (flag‐tagged) with RUVBL2, KAT5, and MYC in HEK293 cells. H) Model of RUVBL1 supporting MYC chromatin binding and target gene expression. Data are represented as mean ± SEM. *P < 0.01 compared to sgCtrl by two‐sided Student's *t*‐test. Source data are available for this figure: SourceData F5 B.

To identify regions of RUVBL1 critical for EwS, we utilized the high‐density CRISPR gene body scan that enables the discovery of functional elements within a protein by saturation mutagenesis achieved through CRISPR‐mediated genome editing.^[^ ^16^ ^]^ We developed a pooled library composed of 194 sgRNAs that target every “NGG” protospacer adjacent motifs (PAM) within the RUVBL1 coding exons (**Figure** **5**A; Figure S1D; targeting density 7.1 bp/sgRNA). We then delivered this RUVBL1 scan library into the A673‐Cas9 cells through the lentiviral transduction and compared the frequencies of each integrated sgRNA sequence before versus after a 16‐d culture using high‐throughput sequencing (SourceData 5). Using a local smoothen modeling,^[^ ^16c^ ^]^ this high‐resolution genetic screen approach revealed the dependency of EwS cells on the N‐terminal AAA domain region G63 – V135 of RUVBL1 (Figure 5B; dotted box). In addition to the previously studied Walker A motif (G70 – T77; an ATP binding site),^[^ ^25^ ^]^ 3D CRISPR scan analysis pinpointed an uncharacterized critical element lysine 108 (K108; the top depleted residue in the screen) at the center of RUVBL1/RUVBL2 hexameric ring^[^ ^26^ ^]^ to be important for RUVBL1's function (Figure 5C).

We then sought to investigate the role of K108 in RUVBL1 via site‐directed mutagenesis. Substitution of K108 with alanine (K108A; Figure 5D) abolished the function of RUVBL1 in rescuing the A673 cells survival, histone H4 acetylation, and EEF1A1 expression from CRISPRi suppression of the endogenous RUVBL1 (Figure 5E,F; Table S2, Supporting Information). Whereas the interactions between RUVBL1/RUVBL2 and RUVBL1/KAT5 remain unaffected by the K108A mutation in RUVBL1, we observed a drastic reduction of MYC in the RUVBL1‐K108‐containing complex (Figure 5G). Collectively, our study suggests a requirement of RUVBL1's K108 residue in supporting the expression of the MYC‐driven EEF1A1 in EwS (Figure 5H).

### 2.6 Synergistic Targeting RUVBL1 and MYC in EwS

**Figure 6:**
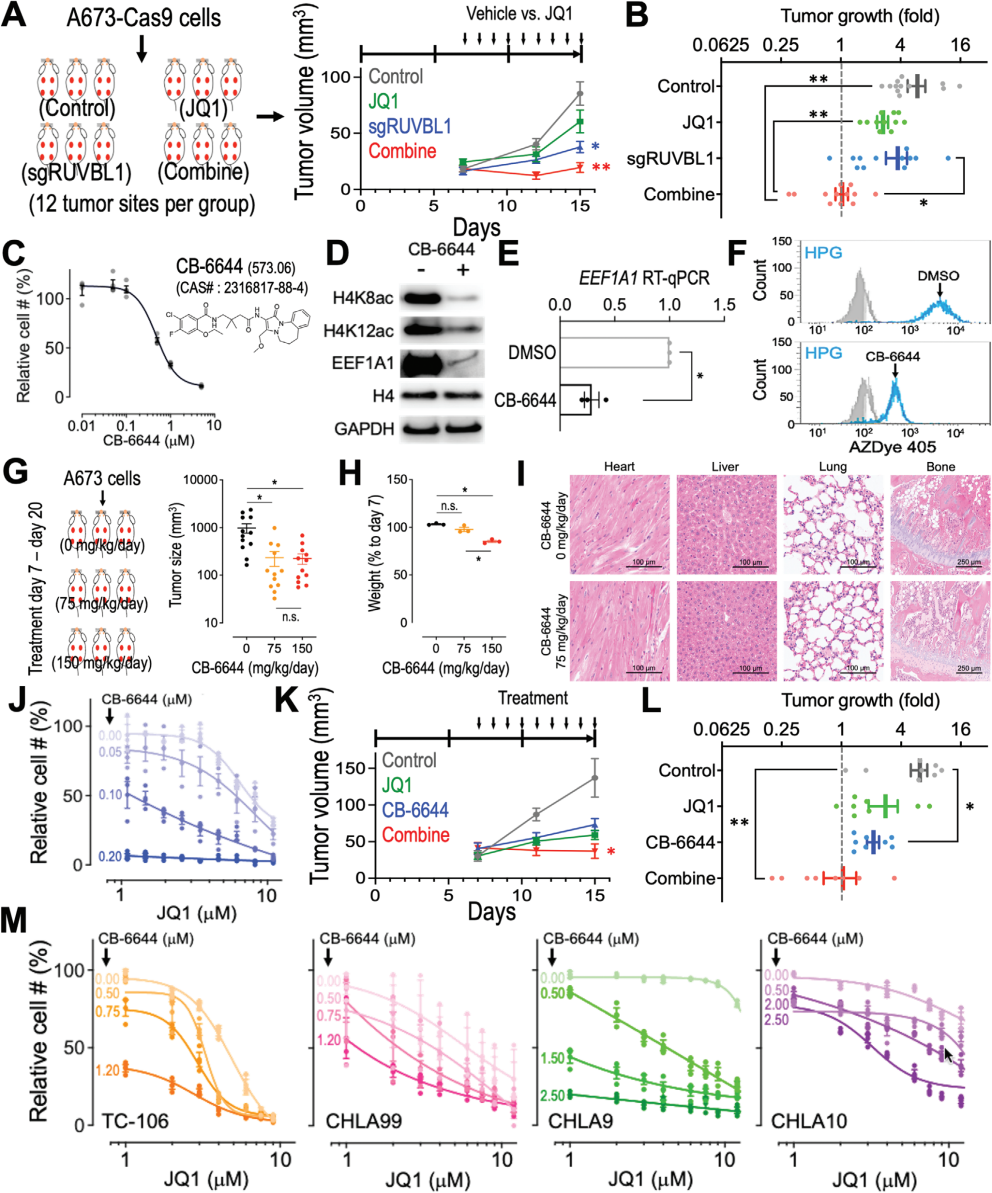
Targeting RUVBL1 synergizes with JQ1 in EwS. A) EwS xenograft tumor volume and B) tumor growth fold (day 15 vs day 7; *n* = 12 tumor sites per group) in control (gray), JQ1 (green), sgRUVBL1 (blue), and combination (red) groups. C) Chemical structure of CB‐6644 and the effect of CB‐6644 treatment on the proliferation of A673 cells (*n* = 4 for each group). D) Western blot of H4K8ac, H4K12ac, EEF1A1, histone H4, and GAPDH in A673 cells treated with vehicle (DMSO) and CB‐6644 (0.5 × 10^−6^ m). E) RT‐qPCR of *EEF1A1* mRNA in A673‐Cas9 cells treated with DMSO and 0.5 × 10^−6^ m CB‐6644 (*n* = 3 for each group). F) Flow cytometric profiles of HPG labeled (cyan) compared to the nonlabeled (gray) cells in A673 cultures incubated with DMSO versus CB‐6644 (0.5 × 10^−6^ m). G) A673 EwS xenograft tumor volume (*n* = 12 tumor sites per group) and H) mouse weight (vs before CB‐6644 treatment; *n* = 3 mice per group) after 14 d of CB‐6644 treatment at 0 (black), 75 (orange), and 150 (red) mg kg^−1^ d^−1^. I) Hematoxylin and eosin stain of the heart, liver, lung, and bone tissues in mice treated with 0 and 75 mg kg^−1^ mL^−1^ of CB‐6644 for 14 d. J) Effect of JQ1 and CB‐6644 combination on the proliferation of A673 cells (*n* = 3 for each condition). Relative cell # (%) of each CB‐6644 condition was normalized to the samples without JQ1 treatment. K) EwS xenograft tumor volume and L) tumor growth fold (day 15 vs day 7; *n* = 8 tumor sites per group) in control (gray), JQ1 (green), CB‐6644 (blue), and combination (red) groups. M) Effect of JQ1 and CB‐6644 combination on the proliferation of patient‐derived Ewing Tumor Family cells TC‐106, CHLA99, CHLA9, and CHLA10 (*n* = 3 for each condition). Data are represented as mean ± SEM. *P < 0.01 and **P < 0.001 compared to control by two‐sided Student's *t*‐test.

Our study revealed a feed‐forward relationship between RUVBL1 and MYC that facilitates the MYC target gene transactivation (Figure 2L). We therefore sought to improve the EwS therapy by combining the sgRUVBL1 with a BET inhibitor JQ1 (a BRD4 bromodomain inhibitor),^[^ ^27^ ^]^ which has been reported to inhibit the expression of MYC through targeting its super‐enhancer^[^ ^28^ ^]^ and demonstrated to suppress EwS tumors in animals.^[^ ^29^ ^]^ Our results showed that, compared to the control tumors (gray), treatment with either JQ1 (green; 40 mg/kg/day) or sgRUVBL1 (blue) could reduce the A673 EwS tumor growth in the NSG xenograft model (**Figure** **6**A,B). Remarkably, the combination of sgRUVBL1 and JQ1 dramatically inhibited the in vivo EwS tumor progression (red; Figure 6A,B), providing a proof‐of‐concept efficacy of synergistic targeting RUVBL1 and MYC in EwS.

In addition to the CRISPR depletion of RUVBL1, we also attempted to enhance the EwS treatment by pharmacologically targeting RUVBL1. While the inhibitors targeting our newly identified MYC‐interacting pocket (centering at RUVBL1‐K108; Figure 5) is currently unavailable, we obtained a pilot inhibitor CB‐6644, which targets the ATP binding pockets in the RUVBL1/2 hexamer.^[^ ^30^ ^]^ We found that CB‐6644 could efficiently suppress the proliferation of A673 cells (Figure 6C) concomitant with reduced histone H4 acetylation, EEF1A1 expression, and protein synthesis (Figure 6D–F), resembling the effect observed from the sgRUVBL1 transduced cells (Figures 3J–L and 4C). In NSG mice, administration of CB‐6644 at 150 mg kg^−1^ d^−1^ was capable of reducing the A673 EwS xenograft tumor growth (Figure 6G); however, this high does CB‐6644 treatment also triggered unfavorable side effects, including a significant weight loss in the treated animals (Figure 6H). On the other hand, 75 mg kg^−1^ d^−1^ of CB‐6644 treatment significantly suppressed the A673 EwS tumor size without affecting the body weight of the recipient mice (Figure 6G,H). Of note, the histological evaluation revealed normal architectures of the cardiac, hepatic, pulmonary, and skeletal (including the growth plate) tissues in the 75 mg kg^−1^ d^−1^ CB‐6644 treated animals (Figure 6I), indicating a potential therapeutic window of in vivo RUVBL1 inhibitory treatment.

Finally, we observed that CB‐6644 increased the sensitivity of A673 cells to JQ1 treatment (i.e., reduced IC50 to JQ1; Figure 6J and Figure S8, Supporting Information) and synergized with JQ1 to suppress the in vivo EwS tumor progression (Figure 6K,L). We also observed comparable synergistic effects between CB‐6644 and JQ1 in a variety of patient‐derived Ewing Tumor Family cells, including the EwS (TC‐106 and CHLA99) and PNET (CHLA9 and CHLA10) samples obtained from the Childhood Cancer Repository (Figure 6M). Our study highlighted the role of RUVBL1 in the oncogenic MYC signaling and pointed to an improved therapeutic strategy via synergistic targeting of the RUVBL1/MYC axis in EwS.

## 3 Discussion and Conclusion 

Similar to the MLL‐fusion oncoproteins that induce malignant leukemia with aberrant epigenetic signatures,^[^ ^31^ ^]^ EwS‐fusion oncoproteins could trigger an oncogenic program through chromatin remodeling^[^ ^6^, ^7^, ^32^ ^]^ and epigenetic reprogramming.^[^ ^6^, ^7^, ^33^ ^]^ A better understanding of the epigenetic dependency in EwS can provide new therapeutic opportunities and shed light on epigenetic mechanisms of mammalian gene regulation. In this study, we performed multiomics analyses including functional genomics (CRISPR library screens), histone modification proteomics (mass spec), transcriptional profiling (RNA‐seq), and chromatin remodeling (ChIP‐seq) analyses in the EwS cells. Using these systems biological approaches, we identified a critical role of the RUVBL1, an ATPase component of the NuA4 histone acetyltransferase complex, in EwS disease progression. We also demonstrated that RUVBL1 contributes to the maintenance of protein synthesis via MYC/KAT5‐driven EEF1A1 expression. Of note, the connection between RUVBL1 and EEF1A1 (also MYC‐to‐EEF1A1 and KAT5‐to‐EEF1A1) for translation control in tumors was not reported before. We further exploited the potential of RUVBL1 inhibition to enhance the efficacy of MYC‐targeted therapy in EwS.

Gene knockdown and knockout screens are powerful genetic approaches to identifying novel effector genes in biological systems.^[^ ^34^ ^]^ Since 2010, multiple RNAi or CRISPR library screens have been performed in EwS and revealed critical mechanisms mediating the EwS maintenance and therapeutic response (summarized in Table S3, Supporting Information).^[^ ^35^ ^]^ Despite these advances, an epigenetic‐focused CRISPR screen (Figure 1A,B) has never been reported in EwS. This is particularly important to EwS as recent studies revealed EwS is amongst the most “mutation cold” tumors,^[^ ^5b,c^ ^]^ arguing a crucial role of the epigenetic mechanisms in EwS etiology and disease progression.^[^ ^35^ ^]^ Secondly, our NuA4 complex‐focused validation screen allowed additional sgRNAs (Figure 1C–E; 25 sgRNAs per gene) and higher statistical confidence to evaluate the role of each NuA4 member in EwS, as compared to typically 4–6 sgRNAs per gene in the genome‐wide CRISPR library.^[^ ^36^ ^]^ In addition, the high‐density RUVBL1 CRISPR tiling scan performed in this study offered a 7.1 bp/sgRNA resolution (Figure 5A–C) and revealed a novel critical residue K108 that is required for RUVBL1/MYC interaction (Figure 5H). Of note, these “serial CRISPR screens” using distinct sgRNA designs (Figure S1B–D, Supporting Information; CRISPRi vs CRISPR‐KO vs CRISPR tiling scan) provided orthogonal evaluations and additional confidence that RUVBL1 is one of the top essential genes in EwS cells. We also envision the structural/functional analysis of the high‐density CRISPR gene scan demonstrated in our study will be highly applicable to other studies in diverse fields.

RUVBL1 and its homolog RUVBL2 belong to the AAA (ATPases Associated with diverse cellular Activities) superfamily that involves in chromatin remodeling, DNA repair, transcription regulation, ribonucleoprotein biogenesis, and mitotic assembly.^[^ ^37^ ^]^ While based on our epigenetic CRISPRi screen (Figure 1A,B), we have focused extensively on the NuA4 complex; nevertheless, the RUVBL1/2 hexamer has been observed in other nucleocomplexes including the SRCAP (also known as the Swr1 in yeast), INO80, and R2TP complexes. For this, we examined the role of the catalytic component of these RUVBL1/2‐containing complexes (KAT5, SRCAP, INO80, and PIH1D1) using CRISPR depletion and the growth competition assay (Figure S9, Supporting Information). Although we cannot exclude the possibility of a cumulative disruption of these additional complexes upon RUVBL1 depletion that also contribute to the essential role of RUVBL1, we observed a significantly stronger dependency of the EwS cells on KAT5 than the catalytic core proteins of the other RUVBL1/2‐containing nucleocomplexes. KAT5 and its orthologue have been implicated as a crucial acetyltransferase mediating the histone acetylation at H4K8 and H4K12 positions in yeast, mouse neurons, human prostate cancer cells, and human dendritic cells.^[^ ^10^, ^12^, ^38^ ^]^ Our study further extended the roles of KAT5 (and its modulator RUVBL1) in maintaining histone H4K8/K12 acetylation in human EwS (Figure 4C,H, and Figure S7, Supporting Information). Finally, it should be noted that the global reduction of the histone H4 acetylation caused by RUVBL1 depletion may have an extended impact on the cancer cells. For instance, we observed the arrested cell cycle, increased apoptosis, together with the impaired protein synthesis in the sgRUVBL1 cells. These cellular stresses could also trigger additional changes in gene expression, histone modification, and chromatin state that are independent of the RUVBL1/MYC/KAT5 pathway examined in this study.

The most well‐characterized functional motifs in RUVBL1 are the Walker A (G70 – T77; ATP binding), Walker B (D302 – H305; ATP hydrolysis), Sensor 1 and 2 (F329 – N332 and T402 – S406; distinguish ADP versus ATP binding), and arginine finger (R357; coordinates the ATP hydrolysis between the subunits in the hexamer).^[^ ^11^, ^25^ ^]^ Aside from these known functional elements in RUVBL1, our CRISPR gene body scan recognized the E105 – L112 region within the N‐terminal AAA domain (G63 – V135; required for MYC interaction)^[^ ^15^, ^39^ ^]^ as the principal element in this protein. This led us to identify the indispensable role of RUVBL1‐K108, a pocket located at the center of the RUVBL1/2 hexameric structure (Figure 5C), in supporting the MYC‐driven gene expression. Of note, this K108 position in RUVBL1 was not revealed in the prior reports that mapped the MYC interaction to another peptide region (residues 136 – 187 of RUVBL1) via chunk deletion (Figure S10A–C, Supporting Information).^[^ ^15a^ ^]^ On the other hand, it has been shown that MYC interacts with the NuA4 complex members, such as the scaffolding protein TRRAP, via its MYC homology box 2 (MB2; aa. 129–145) motif.^[^ ^40^ ^]^ To this end, we found that mutation of K108 residue in RUVBL1 also abolished the interaction between RUVBL1 and TRRAP (Figure S10D, Supporting Information), suggesting that MYC's MB2 motif is likely also involved in its interaction with RUVBL1's K108 pocket. Whether MYC binds to this pocket directly or through an indirect mechanism requires further characterizations; our results pointed to an essential role of RUVBL1's K108 residue for recruiting the NuA4 histone acetyltransferase activity to MYC to assist with its chromatin binding. Furthermore, our CRISPR scan also revealed the Q236 – L240 fragment, a highly conserved region within the less characterized “insertion domain,” to be important for RUVBL1. This notion may encourage further studies aiming to identify additional mechanisms of RUVBL1.

Our current study identified the translation elongation factor EEF1A1 as a novel target of MYC, explaining the pivotal role of MYC in governing the cancer biomass.^[^ ^41^ ^]^ Nevertheless, ectopic expression of EEF1A1 only partially alleviated the impact of sgRUVBL1 on protein synthesis and cell cycle progression (Figure S11, Supporting Information), suggesting additional genes are modulated by the RUVBL1‐MYC axis. Indeed, the MYC high‐affinity binding sites are enriched for ribosomal proteins and cell cycle regulators.^[^ ^42^ ^]^ To this end, we observed reduced expressions of multiple ribosomal components in the sgRUVBL1 transduced cells (Figure S12A,B, Supporting Information), indicating RUVBL1 serves as a critical regulator of ribosomal gene expression. This phenomenon is in line with the reduced MYC chromatin function in the sgRUVBL1 cells. In addition, our RNA‐seq analyses revealed a major role of RUVBL1 in the E2F (an MYC‐regulated transcription factor controlling cell cycle)^[^ ^43^ ^]^ target gene expression (Figure S12C, Supporting Information), indicating the nonribosomal impacts could also be triggered by RUVBL1‐MYC axis.

MYC is an intrinsically disordered protein that consists of an unstructured N‐terminal region (mediates protein interaction and gene transactivation) and a basic helix‐loop‐helix/leucine zipper domain (mediates MYC/MAX DNA binding and dimerization) at the C‐terminal end.^[^ ^44^ ^]^ Lacking a canonical enzymatic pocket, the current MYC targeted therapies rely on disruption of the MYC gene transcription (e.g., JQ1 and other BET inhibitors), translation (e.g., AKT/mTORC inhibitors), protein stability (e.g., USP7 inhibitors), and dimerization (e.g., MYC/MAX blockers).^[^ ^22^, ^45^ ^]^ To this end, our RUVBL1 mechanism provides a critical rationale that the chromatin binding of MYC via epigenetic control could serve as a therapeutic target in cancers. This approach (i.e., through abolishing the MYC chromatin binding to suppress the MYC‐driven gene expression) is distinct from the other MYC targeting strategies and has the potential to combine with the currently available MYC therapeutics for more effective suppression of the oncogenic MYC signaling. Furthermore, while our study extensively focused on EwS, we envision the mechanistic insights and therapeutic opportunities characterized in this project to be applicable to other MYC‐driven malignancies. For example, depletion of RUVBL1 in Ramos cells (a Burkitt lymphoma model characterized by MYC gene translocation and MYC overexpression)^[^ ^46^ ^]^ inhibited the cell proliferation (Figure S13A, Supporting Information) concomitant with reduced levels of H4K8ac, H4K12ac, and EEF1A1 (Figure S13B, Supporting Information). RUVBL1 depletion also decreased the localization of MYC at the *RUVBL1* and *EEF1A1* loci in Ramos cells (Figure S13C, Supporting Information). Reciprocally, depletion of MYC suppressed Ramos cell proliferation (Figure S13D, Supporting Information) and reduced the expression of RUVBL1 and EEF1A1 proteins (Figure S13E, Supporting Information). These results indicated the RUVBL1‐MYC‐EEF1A1 axis observed in EwS is also utilized by Burkitt lymphoma to maintain the oncogenic MYC signaling. Indeed, treatment of the RUVBL1 inhibitor CB‐6644 increased the sensitivity of Ramos cells to JQ1 treatment (i.e., reduced IC50 to JQ1; Figure S13F, Supporting Information), further extending the impact of this combinational therapy beyond EwS.

In summary, our study highlighted that RUVBL1‐mediated chromatin modification is required for oncogenic MYC signaling and EEF1A1‐mediated protein translation. Disruption of RUVBL1 (via genetic suppression or the ATPase inhibition) synergizes with pharmacological targeting of MYC, providing critical rationales toward a more effective combinatorial therapy against EwS and beyond. The insights into the roles of RUVBL1‐K108 in MYC interaction may prompt future efforts aiming to discover novel classes of molecules targeting this pocket. Although the MYC pathways are recognized to play pivotal roles in multiple cancer types, studies on MYC targeted therapy have been focused primarily on inhibiting a single mechanism to disrupt the MYC function. The dynamic interplays between the MYC regulatory network and the therapeutic outcome are just beginning to gain recognition. This study thus represents one of the emerging research fields that explores how the epigenetic mechanisms coordinate in a broad spectrum of biological processes such as transcription factor chromatin targeting, gene expression, protein metabolism, and therapeutic efficacy.

## 4 Experimental Section

### Cell Models and Inhibitors

A673, HEK293, and Ramos cells were obtained from American Type Culture Collection (ATCC). TC‐32 cells were provided by A.J.R. Bishop. TC‐71 cells were obtained from the German Collection of Microorganisms and Cell Cultures (DSMZ). A673 cells were cultured in Dulbecco's modified Eagle medium (DMEM; Gibco) with 10% fetal bovine serum (FBS) (Omega Scientific). TC‐32 cells were cultured in DMEM with 15% FBS. TC‐71 cells were cultured in Iscove's modified Dulbecco's medium (IMDM; Gibco) with 10% FBS. Patient‐derived TC‐106, CHLA99, CHLA9, and CHLA10 cells were cultured in IMDM with 20% FBS and 1× Insulin‐Transferrin‐Selenium (ITS‐G) (51500056, Gibco). Penicillin/streptomycin (Gibco), GlutaMax (Gibco), and plasmocin (0.5 µg mL^−1^; Invivogen) were added to all media. All cells were cultured in 37 °C incubator with 5% CO_2_. Lenti‐dCas9‐KRAB‐Blast (89567, Addgene) and LentiCas9‐Blast (52962, Addgene) expressing cells were established through lentiviral transduction followed by Blasticidin S (10 µg mL^−1^; Gibco) selection, single‐cell cloning, and CRISPR efficiency test (Figure S2, Supporting Information). The RUVBL1/2 inhibitor CB‐6644 was obtained from MedKoo Biosciences (565585). The MYC inhibitor JQ1 was obtained from Millipore/Sigma (SML1524).

### CRISPR Library, cDNA Vectors, and Lentiviral Transduction

For the epigenetics‐focused CRISPR interference (CRISPRi) library, sgiRNA sequences targeting the transcription start site (TSS) of 729 epigenetic‐related genes were selected from the human genome‐wide CRISPRi‐v2 design.^[^ ^47^ ^]^ For the NuA4 complex gene panel and RUVBL1 gene body scan CRISPR libraries, sgRNA sequences targeting the coding regions of the select genes were designed using the Genetic Perturbation Platform (Broad Institute).^[^ ^36b^ ^]^ Briefly, guide RNA oligos were synthesized by microarray (CustomArray) and cloned into the BsmbI sites of the ipUSEPR lentiviral sgRNA vector^[^ ^16^, ^48^ ^]^ that co‐expressed a red fluorescent protein (RFP) and puromycin‐resistance gene (Figure S1A, Supporting Information). To achieve high‐quality CRISPR screens, the cloned sgRNA libraries were first checked by high‐throughput sequencing using a NextSeq550 (Illumina) to ensure a minimum of 90% sgRNAs passed the quality control (Figure S1B–D, Supporting Information). The wild‐type RUVBL1 cDNA was subcloned from an ORF clone (HG14074‐G; Sino Biological) into the pSLIK‐neo (25735, Addgene) lentiviral vector with a C‐terminal flag‐tag. The K108A mutation in RUVBL1 was introduced by site‐directed mutagenesis using PfuUltra II fusion polymerase (600670, Agilent) and QuikChange primers 5′‐CTCAACTGAGATCAAGGCCACAGAGGTGCTGATGG‐3′ and 5′‐CCATCAGCACCTCTGTGGCCTTGATCTCAGTTGAG‐3′. The transduction of pSLIK‐neo lentivirus was selected by geneticin (1 mg mL^−1^; Gibco), and expression of wild‐type and K108A mutant RUVBL1 was induced by adding 1 µg mL^−1^ doxycycline (D9891, Sigma‐Aldrich) in the culture medium. The lentiviral KAT5 and MYC cDNA constructs in the pLenti6.3/V5‐DEST vector (C‐terminal V5‐tag) were obtained from DNASU Plasmid Repository (HsCD00950912 and HsCD00853232). All molecular cloning was performed using the NEB 5‐alpha Competent E. coli (C2987H; New England Biolabs). Lentivirus was produced in HEK293 cells by co‐transfecting the lentiviral plasmids (ipUSEPR, pSLIK‐neo, or pLenti6.3/V5‐DEST systems) with the packaging plasmids psPAX2 (12260, Addgene) and pMD2.G (12259, Addgene). The lentiviral‐containing medium was harvested at 72 h post‐transfection. Virus particles were precipitated in 10% PEG 8000 (BP233‐1, ThermoFisher) at 4 °C for 18 h and then centrifuged at 10 000× *g* for 30 min at 4 °C. The concentrated viral solutions were stored at −80 °C. For lentiviral infection, target cells were mixed with the viral solution and 8 µg mL^−1^ polybrene (TR1003G, MilliporeSigma) and incubated overnight.

### CRISPR Library Screens

The lentivirus of CRISPR libraries was pre‐titrated to obtain ≈15% infection (monitored by flow cytometry for RFP expression) in the A673 cells stably expressing a dCas9‐KRAB (for CRISPRi) or a Cas9 (for CRISPR depletion) construct (Figure S2). Briefly, A673 cells (40 million cells for the epigenetic CRISPRi library screen; 5 million cells for the NuA4 complex CRISPR screen; 3 million cells for the RUVBL1 high‐density CRISPR gene scan) were infected with the CRISPR library and selected by puromycin (1 µg mL^−1^; Gibco). The library‐transduced cells were subcultured every 4 d, and the genomic DNA from the screen samples was collected at the start (day 0) and end (day 16) timepoints. The integrated guide RNA was PCR‐amplified (NEBNext Ultra II Q5; NEB) using DCF01 5′‐CTTGTGGAAAGGACGAAACACCG‐3′ and DCR03 5′‐CCTAGGAACAGCGGTTTAAAAAAGC‐3′ primers and subjected to high‐throughput sequencing using a NextSeq550 (Illumina). To quantify sgRNA reads, 20‐nucleotide sequences that matched the guide RNA backbone structure (5′ prime CACCG and 3′ prime GTTT) were extracted and mapped to the library guide RNA sequences using Bowtie2. The frequency for individual guide RNAs was calculated as the read counts of each sgRNA divided by the total read counts matched to the library. For the epigenetics‐focused CRISPRi screen, the top essential candidate genes were analyzed using the Model‐based Analysis of Genome‐wide CRISPR‐Cas9 Knockout (MAGeCK) algorithm.^[^ ^21^ ^]^ For the NuA4 complex gene panel screen, the CRISPR score was defined as a log10‐fold change in the frequency of individual sgRNAs between the start (day 0) and end (day 16) of the screen samples. For the RUVBL1 CRISPR gene body scan, the CRISPR scan score was defined as a log10‐fold change in the frequency of individual sgRNAs between the start (day 0) and end (day 16) of the screened samples and normalized by the median score of the negative control sgRNA (defined as 0.0; sgRNA targeting nonessential sequences) and the median score of the positive control sgRNA (defined as −1.0; sgRNA targeting *MYC, BRD4, RPA3, PCNA*, etc.) within the screen data. The under‐represented sgRNAs (less than 5% of the average frequency) in the library were excluded from the analysis.

### 3D Protein Structural Annotation of CRISPR Gene Body Scan

The CRISPR scan score of individual sgRNA was first interpolated via Gaussian kernel smoothing in R.^[^ ^16e^ ^]^ Then, the average score over the trinucleotide codons was calculated for each peptide position. Next, 3D structural data of RUVBL1 (PDB ID: 5OAF)^[^ ^26d^ ^]^ were obtained from the Research Collaboratory for Structural Bioinformatics Protein Data Bank (RCSB PDB). Subsequently, the smoothed CRISPR scan scores were mapped onto 3D RUVBL1 structures using the “Defined Attribute” and “Render by Attribute” functionalities in UCSF Chimera 1.15.

### Flow Cytometric Assays

For competition cell culture assays, Cas9‐expressing cells were transduced with the ipUSEPR sgRNA (RFP‐positive) constructs in 96‐well plates at ≈50% infection. Relative RFP% refers to percentages of RFP+ cells over time after lentiviral infection, which was normalized to after 2 d of lentiviral infection. The cell cycle was measured by Click‐iT Plus EdU Alexa Fluor 647 Assay Kits (C10634, Invitrogen). Cells were exposed to 10 × 10^−6^ m EdU at 37 °C for 2 h, and the percentage of cells in the S phase was defined by EdU‐positive cells over the total singlet cells. Cellular apoptosis was detected using CaspGLOW Fluorescein Active Caspase‐3 Staining Kit (88‐7004‐42, Invitrogen). The active caspase 3 in the apoptotic cells was labeled by FITC‐conjugated DEVD‐FMK (a caspase 3 inhibitor). Live cells were defined by 4′,6‐diamidino‐2‐phenylindole (DAPI; D1306, Invitrogen) dye exclusion. Metabolic labeling of the newly synthesized proteins was performed by exposure of cells to l‐azidohomoalanine (AHA; 1066‐25, Click Chemistry Tool) or l‐homopropargylglycin (HPG; 1067‐25, Click Chemistry Tool), followed by AZDye 405 staining of the incorporated AHA (by AZDye 405 DBCO; 1310‐1, Click Chemistry Tool) and HPG (by AZDye 405 Alkyne; 1309‐1, Click Chemistry Tool) using the Click‐&‐Go Cell Reaction Kit (1263, Click Chemistry Tool). Data were obtained by high‐throughput flow cytometry using an Attune NxT flow cytometer with an autosampler (ThermoFisher Scientific).

### Western Blotting and Immunoprecipitation

Cells were lysed in SDS lysis buffer (1% SDS, 50 × 10^−3^ m Tris 7.5) at 95 °C for 10 min, and the protein concentration was determined using DC Protein Assay Kit II (5000112, BioRad). Protein samples were separated electrophoretically using Bolt 4–12% Bis‐Tris plus gels (NW04125BOX, Invitrogen) and transferred onto PVDF membranes (0.2 µm pore size; IB24002, Invitrogen) using iBlot 2 transfer system (Invitrogen). PVDF membranes were immersed in 5% nonfat milk then incubated at 4 °C overnight with primary antibodies against RUVBL1 (HPA019947, Sigma; 1:1000), RUVBL2 (12668S, Cell Signaling Technology; 1:1000), MYC (13987S, Cell Signaling Technology; 1:1000), EEF1A1 (PA5‐17213, Thermo Fisher; 1:1000), KAT5 (12058S, Cell Signaling Technology; 1:1000), H4K8ac (07‐328, Millipore; 1:1000), H4K12ac (61527, Active Motif; 1:1000), histone H4 (ab177840, Abcam; 1:1000), GAPDH (2118S, Cell Signaling Technology; 1:5000), flag‐tag (F7425, Millipore; 1:5000), and V5‐tag (13202S, Cell Signaling Technology; 1:1000). After washing, the membranes were incubated with HRP‐conjugated goat anti‐mouse IgG antibody (31430, Invitrogen; 1:10000) or anti‐rabbit (31460, Invitrogen; 1:10000) at room temperature for 1 hour. Chemiluminescent signals were developed using the Pierce ECL Western Blotting Substrate (32106, Thermo Scientific) and detected using a ChemiDoc imaging system (Bio‐Rad). For immunoprecipitation of RUVBL1, HEK293 cells stably expressing a flag‐tagged wild‐type or K108A RUVBL1 were resuspended in IP lysis buffer (50 × 10^−3^ m Tris PH 7.5, 100 × 10^−3^ m NaCl, 1 × 10^−3^ m EDTA, 1% Triton‐X‐100) supplemented with Halt Protease Inhibitor Cocktail (78430, Thermo Scientific) and incubated on ice for 15 min. The lysates were then centrifuged by 13 000 rpm for 10 min at 4 °C. The flag‐tagged RUVBL1 protein in the supernatants was captured using anti‐FLAG M2 magnetic beads (M8823, Sigma) at 4 °C overnight. After wash, the bead‐captured protein complexes were incubated in SDS lysis buffer (1% SDS, 50 × 10^−3^ m Tris 7.5) at 95 °C for 10 min and then detected by Western blotting. For the input samples, cell lysates of 5 µg total protein each were loaded to the gel. For the IP samples, cell lysate of 100 µg total protein each were used as the starting materials, captured by the anti‐FLAG M2 antibody, and loaded to the gel.

### Ewing Sarcoma Xenograft and Immunohistochemistry

NSG (NOD‐*scid* IL2Rgamma^null^) mice were housed at the animal core facility of City of Hope and used to generate the Ewing sarcoma xenograft model. 6‐ to 8‐week‐old NSG mice were randomly assigned to experimental groups. One million A673 cells transduced with sgCtrl or sgRUVBL1 were resuspended in 100 µL of phosphate‐buffered saline (PBS) and mixed at 1:1 ratio with Matrigel matrix (356234, Corning) for subcutaneous injection in the NSG mice (four tumor sites per mouse). After one week, mice were treated daily with CB‐6644 (75 mg kg^−1^ d^−1^; MedKoo Biosciences), JQ1 (40 mg kg^−1^ d^−1^; S7110, Selleckchem), or vehicle (30% Solutol HS15/PBS) through intraperitoneal injection. Mice were euthanized after the last treatment, and the tumor tissues were collected. All the mouse experiments were approved by the Institutional Animal Care and Use Committee (IACUC) at City of Hope Cancer Center (#17098). For immunohistochemistry, 4‐µm sections of formalin‐fixed and paraffin‐embedded tumor tissues were stained with hematoxylin and eosin for histological evaluation. Apoptotic cells were stained with an anti‐cleaved caspase‐3 (Asp175) antibody (9661, Cell Signaling Technology) using the Ventana Discovery Ultra IHC Auto Staining System (Roche Diagnostics) performed at the City of Hope Pathology Research Service Core.

### Transcriptomic Analysis

For RNA‐seq, total RNA was extracted using RNeasy Mini Kit (74104, QIAGEN) and submitted for mRNA library prep (Novogene) and sequenced by a NovaSeq 6000 (paired‐end 150 bp; ≈20 million reads per sample). Raw sequence reads were mapped to the human genome (GRCh38) using STAR v2.5.3 and calculated using featureCounts v1.5.1. The raw counts were then normalized using the trimmed mean of M values (TMM) method and compared using Bioconductor package “edgeR.” Genes with a minimum average of one read per kilobase per million (RPKM) in the A673 cells were (8600 genes) were selected for analysis. Gene set enrichment analysis (GSEA) was performed using the GSEA v4.0.3 (BROAD Institute). For RT‐qPCR, cDNA was synthesized from 1 µg of extracted total RNA using SuperScript IV First‐Strand synthesis system (18091050, Invitrogen). The qPCR was performed using PowerUp SYBR green master mix (A25742, Applied Biosystems) and a QuantStudio 3 Real‐Time PCR System (Applied Biosystems) with primers listed in Table S4 (Supporting Information).

### Chromatin Immunoprecipitation (ChIP)

Thirty million testing cells were incubated with 1% (v/v) formaldehyde at room temperature for 10 min, followed by adding 125 × 10^−3^ m glycine to quench the excessive formaldehyde. The fixed cells were then washed twice with ice‐cold PBS and resuspended in 250 µL ChIP SDS lysis buffer (1% SDS, 10 × 10^−3^ m EDTA, 50 × 10^−3^ m Tris‐HCl pH 8.0) supplemented with Halt Protease Inhibitor Cocktail (78430, Thermo Scientific). The lysed cells were sonicated by a Bioruptor (Diagenode) to shear the genomic DNA to ≈150–300 bp size, centrifuged at 10 000× *g* for 5 min at room temperature, and the supernatant (contains the sheared chromatin) was collected. For immunoprecipitation, the sheared chromatin samples were mixed with the ChIP dilution buffer (0.01% SDS, 1.1% Triton‐X100, 1.2 × 10^−3^ m EDTA, 167 × 10^−3^ m NaCl, 16.7 × 10^−3^ m Tris‐HCl pH 8.0) at 1:9 ratio, and incubated with the anti‐MYC (13987S, Cell Signaling Technology; 1:400), anti‐H4K8ac (07‐328, Millipore; 1:100), anti‐H4K12ac (61527, Active Motif; 1:100) antibodies at 4 °C for overnight. The antibody‐associated chromatin was then captured by protein A/G magnetic beads (1:400; Dynabeads 10001D and 10003D, Invitrogen) at 4 °C for overnight. For the V5‐tagged KAT5 samples, the sheared chromatin was captured using the anti‐V5‐tag mAb‐magnetic beads (M167‐11, MBL International Corporation) at 4 °C overnight. The magnetic beads were washed with a low salt buffer (0.1% SDS, 1% Triton‐X100, 2 × 10^−3^ m EDTA, 150 × 10^−3^ m NaCl, 20 × 10^−3^ m Tris‐HCl pH 8.0) followed by a high salt buffer (0.1% SDS, 1% Triton‐X100, 2 × 10^−3^ m EDTA, 500 × 10^−3^ m NaCl, 20 × 10^−3^ m Tris‐HCl pH 8.0), a LiCl wash buffer (250 × 10^−3^ m LiCl, 1% IGEPAL‐CA630, 1% deoxycholic acid, 1 × 10^−3^ m EDTA, 10 × 10^−3^ m Tris‐HCl pH 8.0), and the TE buffer (1 × 10^−3^ m EDTA, 10 × 10^−3^ m Tris‐HCl pH 8.0). The washed beads were then incubated with reverse‐crosslinking buffer (1.1% SDS, 110 × 10^−3^ m sodium bicarbonate) at 65 °C overnight, followed by GeneJET DNA purification (K0702, Thermo Scientific). The ChIP enriched genomic DNA was detected by qPCR of the *EEF1A1* or *RUVBL1* locus (primers listed in Table S5, Supporting Information) and normalized to the input genomic DNA (without ChIP enrichment). For MYC ChIP‐seq, the input and anti‐MYC antibody‐captured genomic DNA samples were submitted for library prep and NovaSeq 6000 sequencing (paired‐end 150 bp reads; ≈50 million reads per sample). The raw sequence reads were quality checked using the FASTQC software (version 0.11.8) and aligned against the human genome hg38 using Burrows‐Wheeler Aligner (version 0.7.17). The aligned reads were then sorted by Samtools (version 1.10) and the duplicated reads were removed by Picard MarkDuplicates (version 2.21.1). Peak‐calling analysis to identify antibody‐binding regions was performed using MACS2 (version 2.1.1) and the SPMR option was used to generate normalized pileup files for downstream analysis. ChIP‐seq signals were calculated from the pileup files around TSS regions and visualized in plots using deepTools (version 3.3.0). Genes with more than tenfold enrichment of MYC ChIP‐seq signal over input at their TSS ± 1 kb regions were selected as MYC targets (1741 genes).

### Mass Spectrometric Analysis of Histone Acetylation

Five million sgCtrl or sgRUVBL1 transduced A673 cells were harvested, washed once with PBS, and spun down at 500× *g* for 5 min. The cell pellets were flash‐frozen with dry ice and submitted for Mod Spec Service (Active Motif). Briefly, histones were acid extracted, derivatized via propionylation, and digested with trypsin. The newly formed N‐termini were then propionylated, and the tryptic peptide samples were measured with three technical replicates using the TSQ Quantum Ultra mass spectrometer coupled with an UltiMate 3000 Dionex nano‐liquid chromatography system (Thermo Scientific). The data were quantified using the Skyline.^[^ ^49^ ^]^ The acetylation positions on histone H3/H4 that exhibit more than 0.1% of total histone were reported.

### Code Availability 

The computational codes/tool packages used in this study include Genetic Perturbation Platform (Broad Institute,^[^ ^36b^ ^]^ Bowtie2,^[^ ^50^ ^]^ MAGeCK,^[^ ^21^ ^]^ Gaussian kernel smoothing in R,^[^ ^51^ ^]^ UCSF Chimera 1.15,^[^ ^52^ ^]^ Attune NxT v3.1.2 (ThermoFisher), STAR v2.5.3,^[^ ^53^ ^]^ featureCounts v1.5.1,^[^ ^54^ ^]^ edgeR,^[^ ^55^ ^]^ GSEA v4.0.3 (BROAD Institute),^[^ ^56^ ^]^ FASTQC software (version 0.11.8), Burrows‐Wheeler Aligner (version 0.7.17), Samtools (version 1.10), Picard MarkDuplicates (version 2.21.1), MACS2 (version 2.1.1), deepTools (version 3.3.0), Skyline,^[^ ^49^ ^]^ IGV 2.11.0 (Broad Institute), BioRender (https://biorender.com), QuantStudio Design & Analysis Software v1.5.1 (Applied Biosystems), and Bio‐Rad ChemiDoc MP (Bio‐Rad). Two‐sided Student's *t*‐test was carried out using Prism 9 (GraphPad) to determine the statistical significance of difference between variables.

### Availability of Materials

Cas9‐expressing A673 cells, K108A‐RUVBL1 cDNA, and CRISPR libraries for epigenetic regulators, NuA4 complex, and RUVBL1 will be available upon request. All other biological materials are commercially available.

### Statistical Analysis

Data are represented as mean ± SEM. *P* < 0.01 was considered as statistically significant; *P < 0.01 and **P < 0.001 compared to control. Using two‐tailed unpaired *t*‐test, the differences between every two groups were analyzed. Statistics was performed by GraphPad Prism 9.

## Conflict of Interest

J.C. is a scientific founder of Genovel Biotech Corp. and holds equities with the company, and is also a Scientific Advisor for Race Oncology.

## Author Contributions

M.L., A.K.N.C., S.P.P., N.M., Q.L., X.X., W.‐H.C., K.M., P.S., L.Z., M.L., J.W., J.W., B.C., L.N.C., J.L., and X.H.Z. performed the experiments; M.L., L.Y., and C.‐W.C. analyzed the data; S.T.R., M.M, J.C., J.Q., K.H., A.J.R.B., and C.‐W.C. provided conceptual input; M.L., L.Y., and C.‐W.C. wrote the paper; C.‐W.C. conceived and supervised the study.

## Supporting information

Supporting Information

Supporting Information

## Data Availability

The data that support the findings of this study are openly available in Gene Expression Omnibus (GEO) at https://www.ncbi.nlm.nih.gov/geo/query/acc.cgi?acc=GSE182378, reference number 182378. 3D protein structure (PDB ID 5OAF) was obtained from the Research Collaboratory for Structural Bioinformatics Protein Data Bank (RCSB PDB; https://www.rcsb.org).^[57]^ The data that support the findings of this study are available from the corresponding author upon reasonable request.
