# Correction to “Epigenetic Control of Translation Checkpoint and Tumor Progression via RUVBL1‐EEF1A1 Axis”

**DOI:** 10.1002/advs.202519908

**Published:** 2025-11-06

Li, M., L. Yang, A. K. N. Chan, S. P. Pokharel, Q. Liu, N. Mattson, X. Xu, et al. “Epigenetic Control of Translation Checkpoint and Tumor Progression Via RUVBL1‐EEF1A1 Axis.” *Advanced Science* (Apr 19, 2023): e2206584. https://doi.org/10.1002/advs.202206584.

The authors admitted to an image compilation error in the subpanels of Figure 4I and were able to provide the original images. Additionally, the Western blots for RUVBL1, H4, and GAPDH in Figure 4C were the same blots to those presented in Figure 2E. The authors confirm that all the experimental results and corresponding conclusions mentioned in the paper remain unaffected. The corrected Figure 4 is shown as follows.

Corrected Figure 4


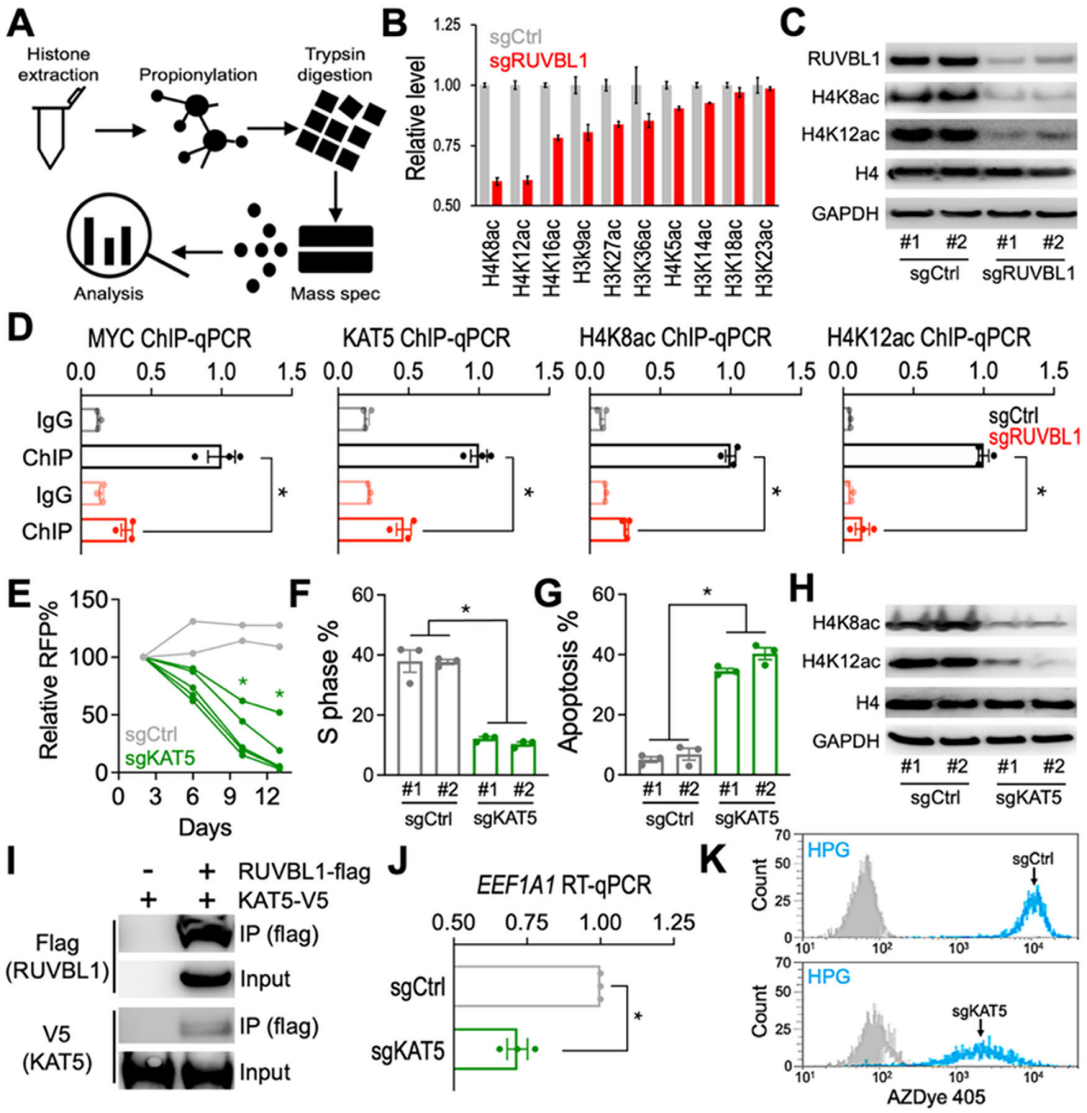


We apologize for this error.

