# Supplementary material for: Epigenetic Control of Translation Checkpoint and Tumor Progression via RUVBL1‐EEF1A1 Axis
Source: Adv Sci (Weinh). 2023 Apr 19;10(17):2206584. doi: 10.1002/advs.202206584 (PMC10265057; doi:10.1002/advs.202206584)
Supplement: Supplementary file 1 — Supporting Information [file ADVS-10-2206584-s001.pdf]

ADVANCED SCIENCE
Open Access

Supporting Information

for *Adv. Sci.*, DOI 10.1002/advs.202206584

Epigenetic Control of Translation Checkpoint and Tumor Progression via RUVBL1-EEF1A1 Axis

*Mingli Li, Lu Yang, Anthony K. N. Chan, Sheela Pangeni Pokharel, Qiao Liu, Nicole Mattson, Xiaobao Xu, Wen-Han Chang, Kazuya Miyashita, Priyanka Singh, Leisi Zhang, Maggie Li, Jun Wu, Jinhui Wang, Bryan Chen, Lai N. Chan, Jaewoong Lee, Xu Hannah Zhang, Steven T. Rosen, Markus Müschen, Jun Qi, Jianjun Chen, Kevin Hiom, Alexander J. R. Bishop and Chun-Wei Chen**

Supporting Information

Epigenetic control of translation checkpoint and tumor progression via RUVBL1-EEF1A1 axis

Mingli Li[1], Lu Yang[1,2], Anthony K.N. Chan[1,2], Sheela Pangeni Pokharel[1,2], Qiao Liu[1], Nicole Mattson[1], Xiaobao Xu[1], Wen-Han Chang[1], Kazuya Miyashita[1], Priyanka Singh[1], Leisi Zhang[1], Maggie Li[1], Jun Wu[3], Jinhui Wang[3], Bryan Chen[1], Lai N. Chan[4], Jaewoong Lee[4,5,6], Xu Hannah Zhang[3], Steven T. Rosen[3], Markus Müschen[4], Jun Qi[7], Jianjun Chen[1,3], Kevin Hiom[8], Alexander J.R. Bishop[9,10], Chun-Wei David Chen[1,2,3,#]

Affiliations:

[1]Department of Systems Biology, Beckman Research Institute, City of Hope, Duarte, CA, USA

[2]Division of Epigenetic and Transcriptional Engineering, Beckman Research Institute, City of Hope, Duarte, CA, USA

[3]City of Hope Comprehensive Cancer Center, Duarte, CA, USA

[4]Center of Molecular and Cellular Oncology, Yale Cancer Center, Yale School of Medicine, New Haven, CT, USA

[5]School of Biosystems and Biomedical Sciences, College of Health Science, Korea University, Seoul, South Korea

[6]Interdisciplinary Program in Precision Public Health, Korea University, Seoul, South Korea

[7]Department of Cancer Biology, Dana-Farber Cancer Institute, Harvard Medical School, Boston, MA, USA

[8]Division of Cellular Medicine, School of Medicine, University of Dundee, Scotland, UK.

[9]Department of Cellular Systems and Anatomy, University of Texas Health Science Center at San Antonio, San Antonio, Texas, USA

[10]Greehey Children's Cancer Research Institute, University of Texas Health Science Center at San Antonio, San Antonio, Texas, USA

Figure S1 to S13

Table S1 to S5

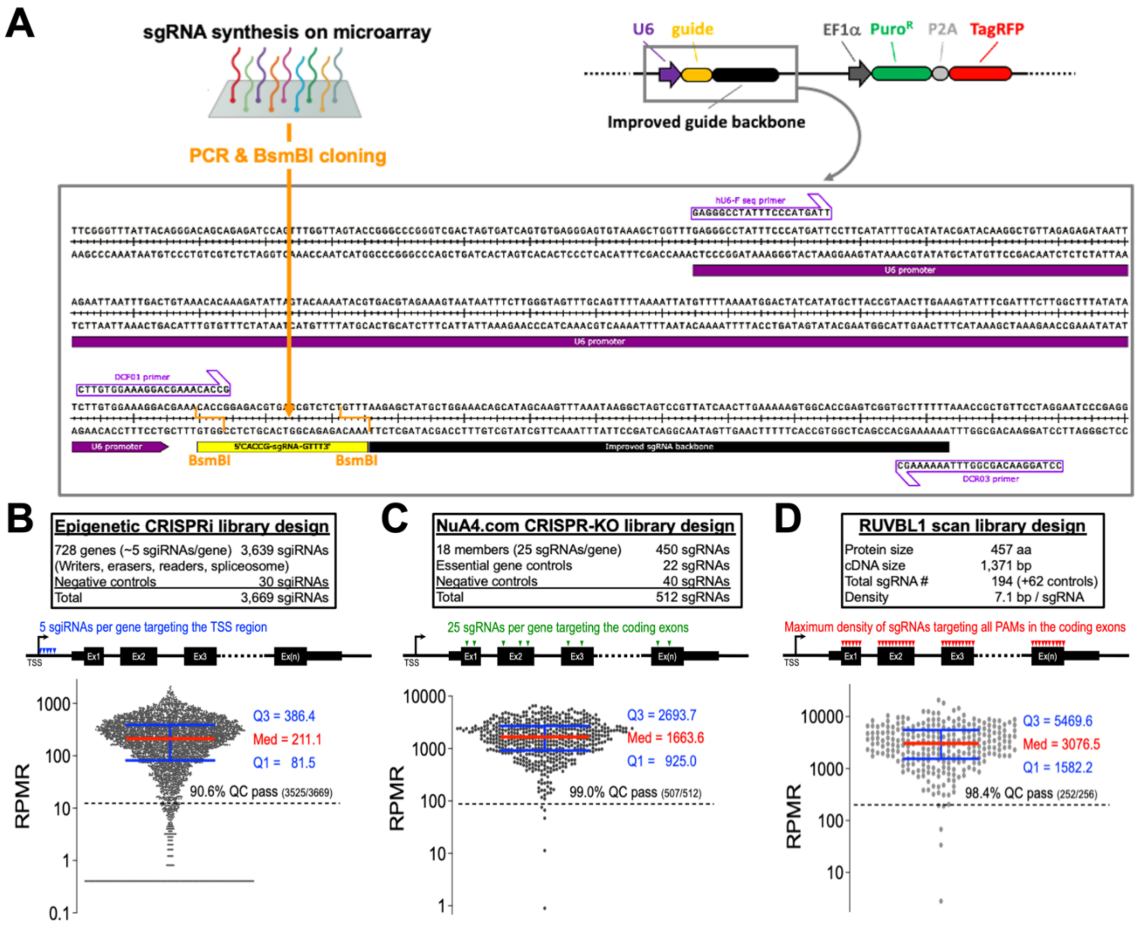

Figure S1. CRISPR genetic screen libraries used in this study.

(A) Map of the ipUSEPR vector expressing a sgRNA together with a puromycin-resistant gene (PuroR) and a TagRFP fluorescent protein. Primers for Sanger (hU6-F_seq) and Illumina (DCF01 and DCR03) sequencing are listed. (B-D) Design and distribution of individual sgRNA frequencies RPMR (reads per million reads) in the CRISPR libraries targeting (B) epigenetic-related genes, (C) NuA4 complex members, and (D) coding regions of *RUVBL1*. (B) 90.6%, (C) 99.0%, and (D) 98.4% of sgRNA in these libraries passed the QC by exhibiting ≥ 5% of average frequency.

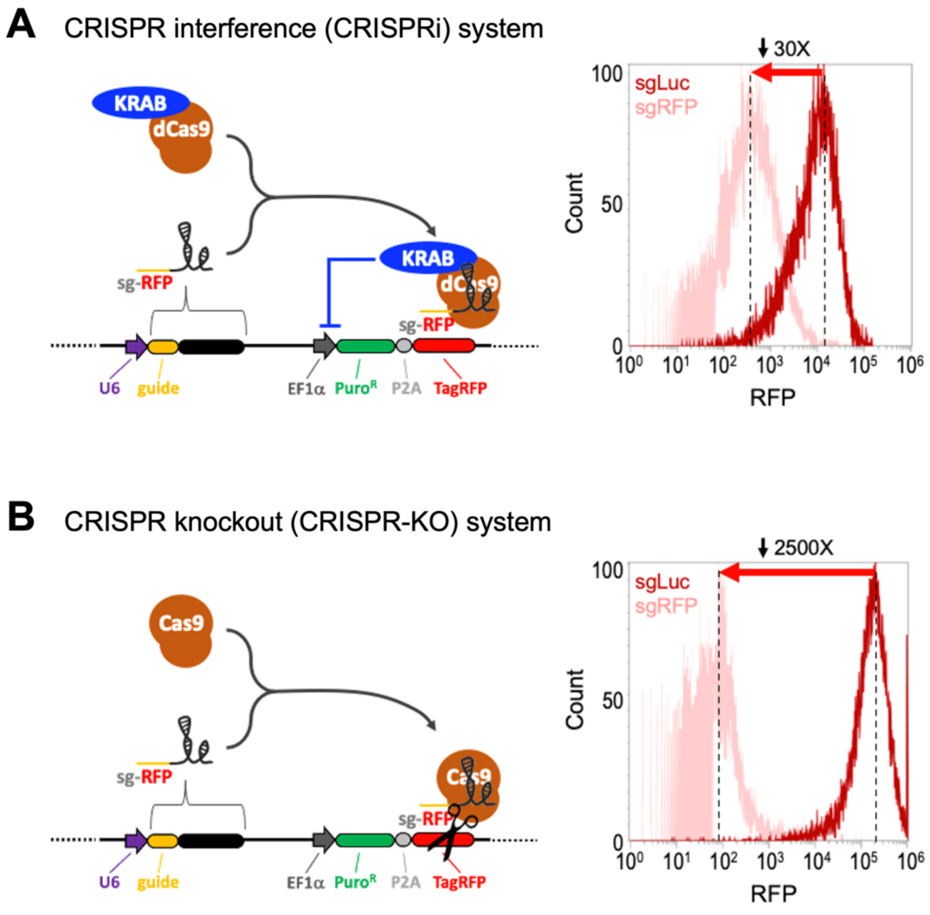

Figure S2. Validation of CRISPR efficiency using an RFP inactivation assay.
(A) CRISPR interference and (B) CRISPR knockout cell systems used in this study. The reduced expression of EF1α-driven RFP in the ipUSEPR vector serves as a reporter of CRISPR editing efficiency.

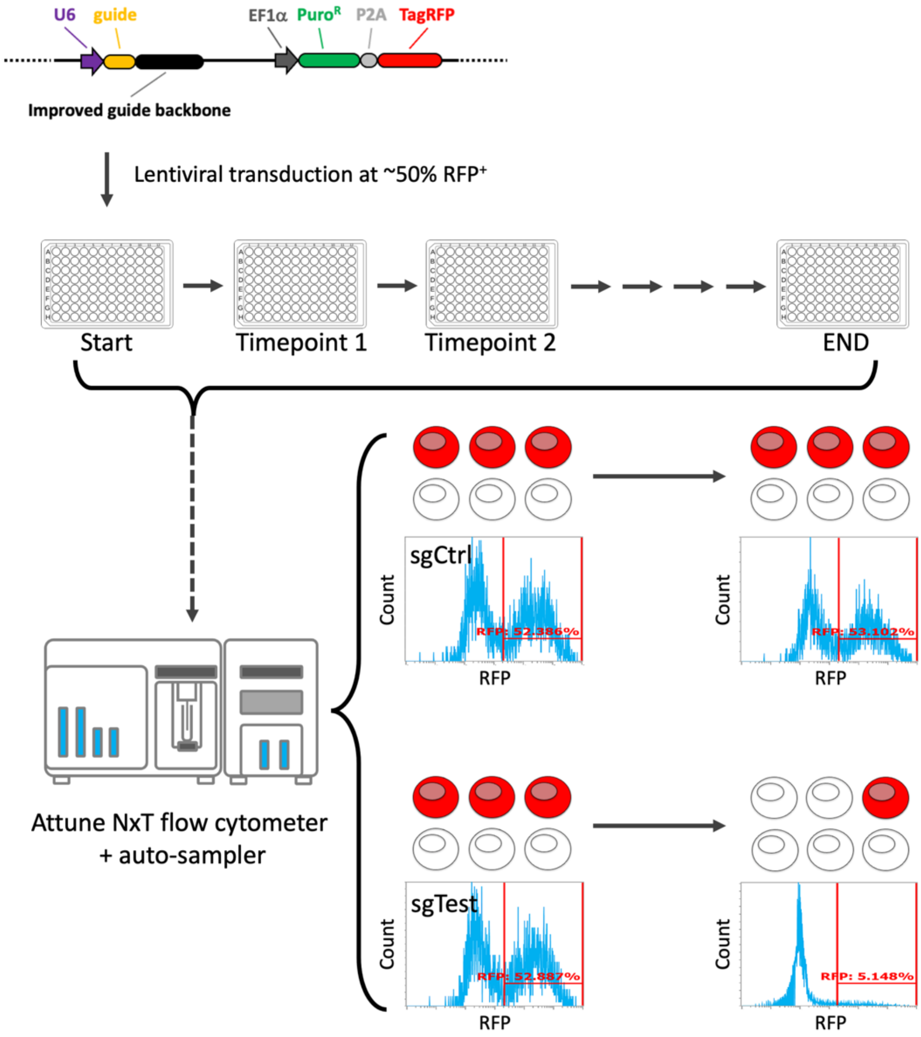

Figure S3. Schematic outline of the RFP flow cytometric growth competition assay.

Source Name	Patient ID	RUVBL1 exp.	Age	Diagnosis	EFS (months)	OVS (months)	Gender	Status
GSM439907	R52	1.317	22	Ewing	14.4	23.2	F	dead
GSM439914	R61	1.289	15	Ewing	16.2	16.2	M	NED
GSM439906	R51	1.266	24	Askin	4.6	10.9	M	dead
GSM439893	R37	1.254	11	Ewing	9.8	9.8	M	dead
GSM439911	R57	1.252	11	Ewing	2	8.7	M	dead
GSM439900	R44	1.249	30	Ewing	19.8	25.6	F	dead
GSM439922	R74	1.239	27	PNET	110.4	110.4	M	NED
GSM439912	R58	1.237	22	Ewing	13.4	13.4	M	dead
GSM439896	R40	1.219	24	Ewing	18.2	117.3	M	NED
GSM439913	R60	1.216	12	Ewing	121.5	121.5	M	NED
GSM439890	R33	1.212	15	Ewing	16.6	16.6	M	dead
GSM439892	R35	1.211	16	Ewing	10	13.6	F	dead
GSM439905	R50	1.206	16	PNET	58.3	99.3	M	dead
GSM439916	R63	1.187	13	Ewing	43.4	43.4	M	NED
GSM439929	R84	1.173	9	Ewing	27.9	41.6	M	dead
GSM439899	R43	1.172	17	Ewing	24.8	35.7	F	dead
GSM439927	R81	1.152	24	Ewing	34.4	81.4	M	dead
GSM439901	R45	1.144	15	Askin	17.9	24.6	M	dead
GSM439910	R55	1.138	16	PNET	6.8	8.5	F	dead
GSM439902	R46	1.135	7	PNET	22.1	64.2	F	dead
GSM439918	R65	1.131	20	Ewing	11	11	F	dead
GSM439894	R38	1.131	23	Ewing	11.3	11.3	M	dead
GSM439924	R78	1.128	11	PNET	69.5	69.5	F	NED
GSM439888	R29	1.128	21	Ewing	4.6	4.6	M	dead
GSM439897	R41	1.115	15	Ewing	125.1	125.1	M	NED
GSM439904	R49	1.115	34	Ewing	18.6	126.2	M	NED
GSM439903	R48	1.111	17	Ewing	31.6	46.2	M	dead
GSM439898	R42	1.110	18	Ewing	192.2	192.2	M	NED
GSM439915	R62	1.105	16	Ewing	117.6	117.6	M	NED
GSM439926	R80	1.100	8	PNET	23.9	68.5	F	NED
GSM439928	R83	1.097	4	PNET	70.1	129.1	F	NED
GSM439920	R69	1.095	9	Ewing	62.6	62.6	F	NED
GSM439895	R39	1.083	16	Ewing	47	47	F	dead
GSM439909	R54	1.076	22	Ewing	25.9	61.5	M	dead
GSM439925	R79	1.057	21	PNET	127.1	127.1	M	NED
GSM439921	R72	1.054	32	PNET	31.5	117.7	F	NED
GSM439908	R53	1.038	19	Ewing	87.7	87.7	F	NED
GSM439917	R64	1.002	25	Ewing	15.3	21.3	M	dead
GSM439919	R67	0.988	14	Ewing	16.7	29.4	M	dead
GSM439887	R196	0.975	5	Ewing	14	20.7	M	dead
GSM439886	R194	0.952	32	Ewing	11.7	15.6	F	dead
GSM439889	R30	0.919	26	Ewing	28.6	48.9	F	NED
GSM439923	R75	0.918	14	PNET	9.8	25.4	M	dead
GSM439891	R34	0.815	18	Ewing	19.4	56.5	M	AWD

Ewing: Ewing sarcoma; Askin: Askin tumor; PNET: Primitive neuroectodermal tumor
EFS: Event free survival; OVS: Overall survival
NED: No evedence of disease; AW: Alive with disease

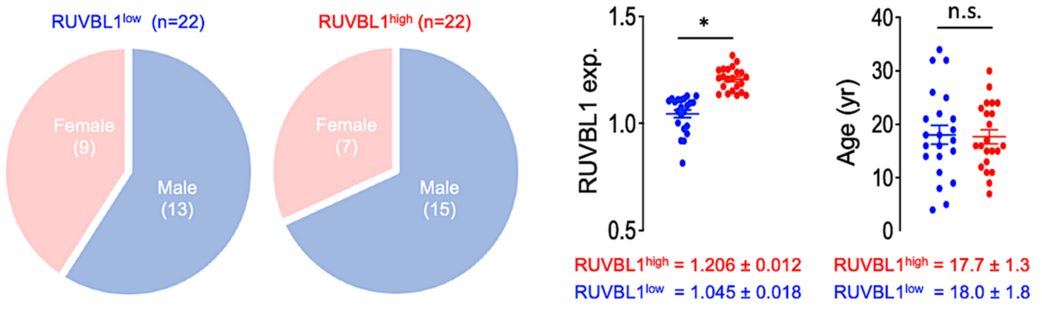

Figure S4. Information of patients with Ewing sarcoma family of tumors.
Source: EMBL-EBI (E-GEOD-17618)

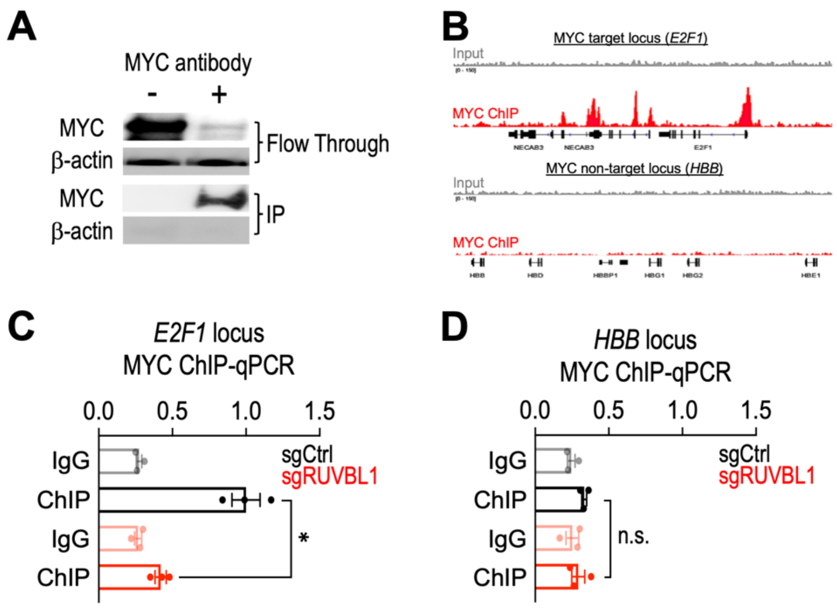

Figure S5. Evaluation of MYC ChIP-seq in EwS cells.

(A) Efficient capture of MYC protein using a rabbit mAb D3N8F (13987S, Cell Signaling Technology; 1:400). (B) Profiles of MYC ChIP-seq at *E2F1* (E2F Transcription Factor 1; an MYC target locus) and *HBB* (Hemoglobin Subunit Beta; an MYC non-target locus) loci in A673 cells. MYC ChIP-qPCR at (C) *E2F1* and (D) *HBB* loci in A673 cells transduced with sgCtrl and sgRUVBL1. Data are represented as mean ± SEM. *P < 0.01 compared to sgCtrl by two-sided Student's t-test.

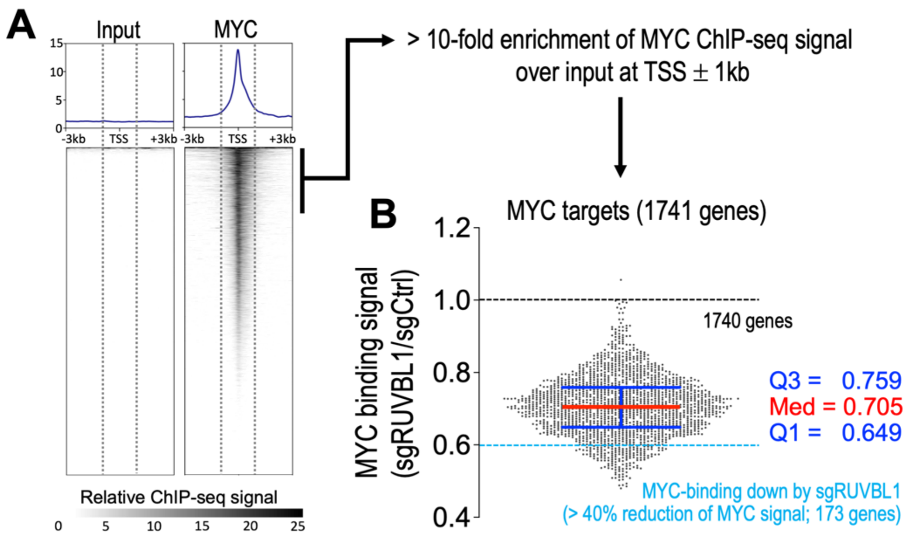

Figure S6. Evaluation of MYC chromatin binding targets.

(A) MYC ChIP-seq signal for each gene was calculated from the pileup files around TSS ± 1kb regions. Genes with more than 10-fold enrichment of MYC ChIP-seq signal in the MYC antibody captured sample over the input sample (without ChIP enrichment) were selected as MYC targets (1741 genes). (B) Depletion of RUVBL1 led to reduced MYC binding signals in the majority of MYC targets (median ratio 0.705), suggesting that RUVBL1 serves as a master regulator of MYC's chromatin binding.

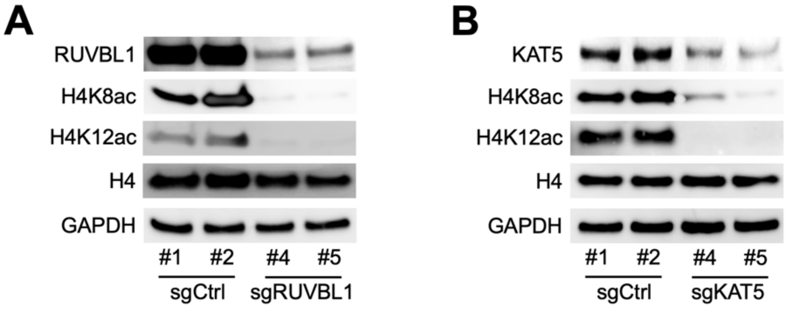

Figure S7. Validation of the effects of sgRUVBL1 and sgKAT5 on histone H4 acetylation.
(A) Western blot of RUVBL1, H4K8ac, H4K12ac, histone H4, and GAPDH in A673-Cas9 cells transduced with sgCtrl (#1 and #2) vs. sgRUVBL1 (#4 and #5). (B) Western blot of KAT5, H4K8ac, H4K12ac, histone H4, and GAPDH in A673-Cas9 cells transduced with sgCtrl (#1 and #2) vs. sgKAT5 (#4 and #5). The guide RNA sequences are listed in Table S1.

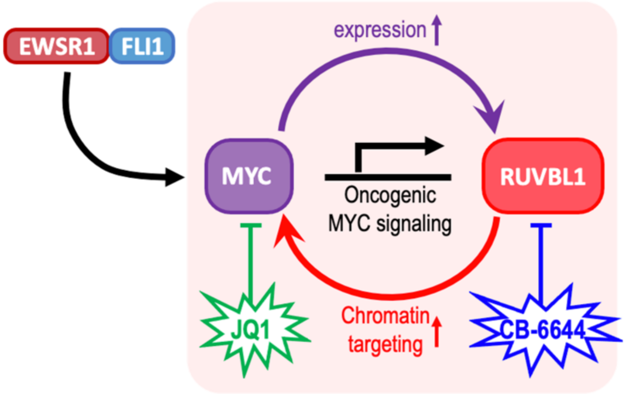

Figure S8. Model of combinational targeting the RUVBL1/MYC feed-forward network by CB-6644 and JQ1 in EwS.

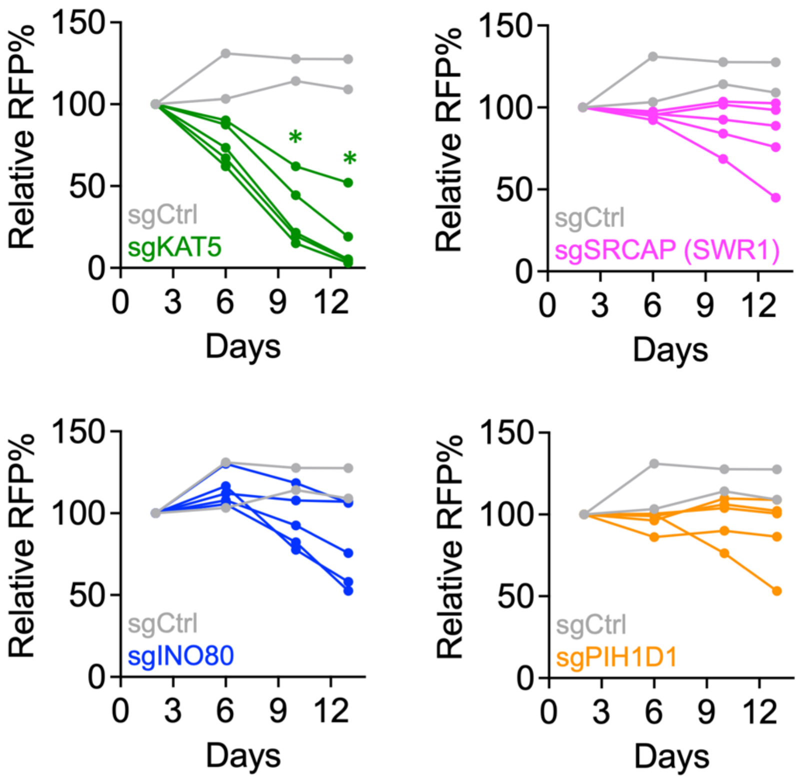

Figure S9. Roles of KAT5, SRCAP, INO80, and PIH1D1 in A673 cells.
Growth competition assay of sgCtrl (grey lines; n = 2 independent sgRNA sequences), sgKAT5 (a NuA4 complex member; green lines; n = 5 independent sgRNA sequences), sgSRCAP (a SWR1 complex member; pink lines; n = 5 independent sgRNA sequences), sgINO80 (an INO80 complex member; blue lines; n = 5 independent sgRNA sequences), and sgPIH1D1 (an R2TP complex member; orange lines; n = 5 independent sgRNA sequences) in A673-Cas9 cells. *P < 0.01 compared to sgCtrl by two-sided Student's t-test.

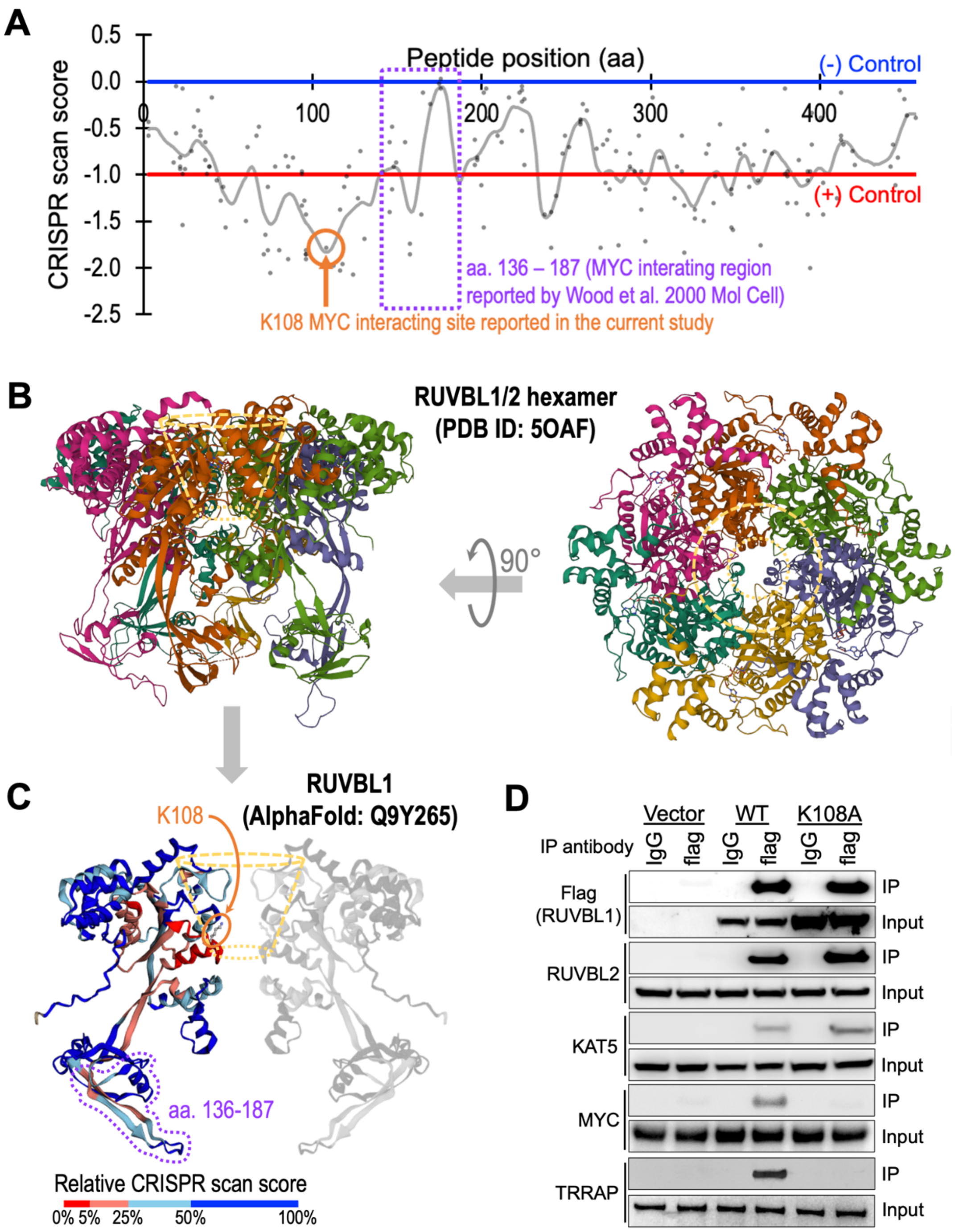

Figure S10. Comparison of the MYC interacting regions in RUVBL1 reported by different studies.
(A) Two-dimensional annotation of the MYC binding regions (K108 vs. aa. 136 – 187) relative to the RUVBL1 CRISPR gene body scan profile. (B) Side (left) and top (right) views of the RUVBL1/2 hexamer cryo-EM structure (PDB ID: 5OAF). (C) Three-dimensional annotation of the CRISPR scan score and MYC binding regions (K108 vs. aa. 136 – 187) relative to an AlphaFold model of RUVBL1 (Q9Y265). The RUVBL1/2 hexamer central pocket is highlighted (yellow dashed pocket). (D) Co-IP of WT- and K108A-RUVBL1 (flag-tagged) with RUVBL2, KAT5, MYC, and TRRAP in HEK293 cells.

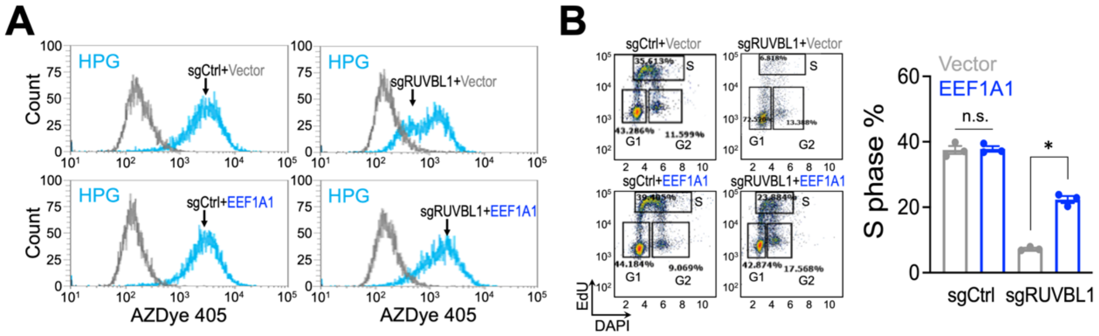

Figure S11. Expression of EEF1A1 partially rescues the RUVBL1-depleted EwS cells.
(A) Effect of EEF1A1 cDNA expression on flow cytometric profiles of HPG labeled (cyan) compared to the non-labeled (grey) cells in A673 cultures transduced with sgCtrl and sgRUVBL1. (B) Effect of EEF1A1 cDNA expression on cell cycle monitored by EdU incorporation. Data are represented as mean ± SEM. *$P < 0.05$ by two-sided Student's t-test.

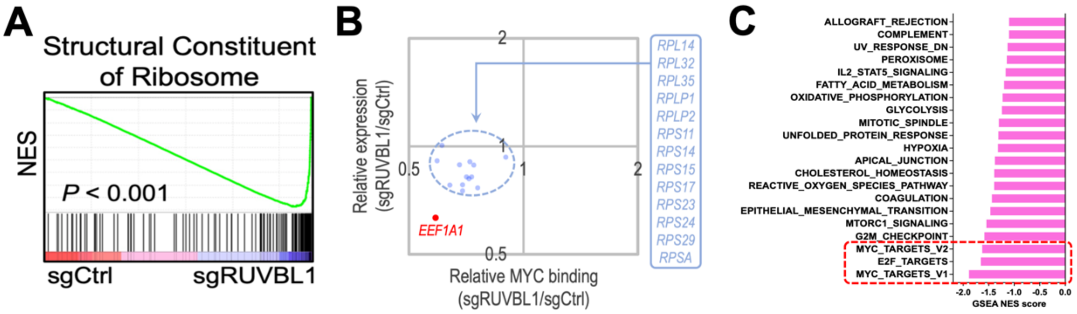

Figure S12. Effect of RUVBL1-depletion on gene expression.

(A) RNA-seq and GSEA analysis showing a reduced expression of the "Structural Constituent of Ribosome" gene set in the RUVBL1 depleted A673 cells. (B) *EEF1A1* (red dot) exhibited a more pronounced reduction in both MYC binding and expression levels upon RUVBL1 depletion than the other 13 MYC-targeted ribosomal genes (blue dots). (C) RNA-seq and GSEA analysis showing top depleted Hallmark gene sets in the sgRUVBL1 transduced A673 cells.

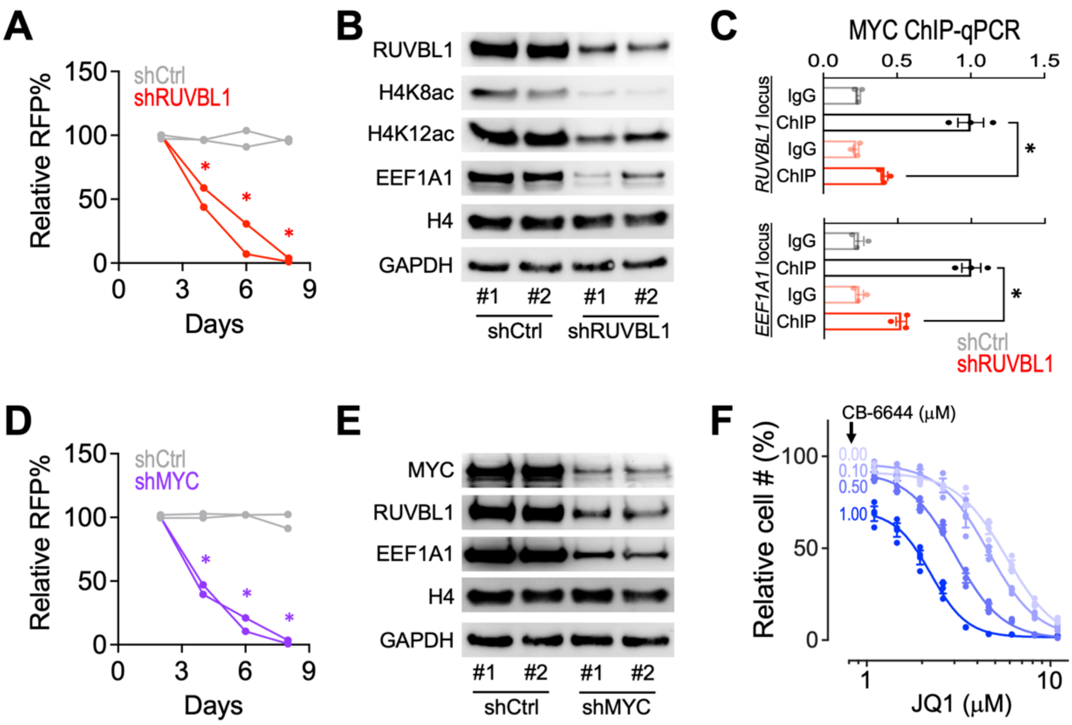

G

shRNA_ID	Sequence	shRNA_ID	Sequence	shRNA_ID	Sequence
shCtrl#1	GATTCTAAAACGGATTACCA	shRUVBL1#1	GTGGCGTCATAGTAGAATTAA	shMYC#1	CCCAAGGTAGTTATCCTTAAA
shCtrl#2	GGATGATAACTGGTCCGCAG	shRUVBL1#2	CCGGCCAACTTGCTTGCTAAA	shMYC#2	ACTGAAAGATTTAGCCATAAT

Figure S13. RUVBL1-MYC-EEF1A1 axis in Burkitt lymphoma cells.

(A) Growth competition assay of Ramos cells transduced with RFP-labeled shCtrl (grey lines; n = 2 independent shRNA sequences) and shRUVBL1 (red lines; n = 2 independent shRNA sequences). (B) Western blot of RUVBL1, H4K8ac, H4K12ac, EEF1A1, histone H4, and GAPDH in Ramos cells transduced with shCtrl vs. shRUVBL1 (2 independent shRNA sequences per group). (C) MYC ChIP-qPCR at the *RUVBL1* and *EEF1A1* loci in Ramos cells transduced with shCtrl and shRUVBL1 (n = 3 for each group). (D) Growth competition assay of Ramos cells transduced with RFP-labeled shCtrl (grey lines; n = 2 independent shRNA sequences) and shMYC (purple lines; n = 2 independent shRNA sequences). (E) Western blot of MYC, RUVBL1, EEF1A1, histone H4, and GAPDH in Ramos cells transduced with shCtrl vs. shMYC (2 independent shRNA sequences per group). (F) Effect of JQ1 and CB-6644 combination on the proliferation of Ramos cells (n = 3 for each condition). Relative cell # (%) of each CB-6644 condition was normalized to the samples without JQ1 treatment. (G) Sequences of shCtrl, shRUVBL1, and shMYC used in this study. Data are represented as mean ± SEM. *P < 0.01 compared to shCtrl by two-sided Student's t-test.

Table S1. CRISPR-KO sgRNA sequences.

sgRNA_ID	Sequence
sgCtrl#1	GATTCTAAAACGGATTACCA
sgCtrl#2	GGATGATAACTGGTCCGCAG
sgRUVBL1#1	ACTTGGATGTGGCTAATGCG
sgRUVBL1#2	GGAAGGAATCAACATCAGTG
sgRUVBL1#3	GTGAACAAGTACATCGACCA
sgRUVBL1#4	TGGTCCGGATTATCATCACT
sgRUVBL1#5	CATCAACAAACAGCACACCC
sgKAT5#1	GGGTACGGGGAGAAGTACCA
sgKAT5#2	ACTTGAGGCAGAACTCGCAC
sgKAT5#3	AGGCAATGAGATTTACCGCA
sgKAT5#4	AACACTTGGCCAAAAGACAC
sgKAT5#5	GATTGATGGACGTAAGAACA
sgMYC#1	CTATGACCTCGACTACGACT
sgMYC#2	AGAGTGCATCGACCCCTCGG
sgMYC#3	CTTCGGGGAGACAACGACGG
sgMYC#4	CGAGGAGAGCAGAGAATCCG
sgMYC#5	CCAGAGTTTCATCTGCGACC
sgEEF1A1#1	ATTACAGGGACATCTCAGGT
sgEEF1A1#2	CATCTGATCTATAAATGCGG
sgEEF1A1#3	TTAATCCTTACAGATGGGAA
sgEEF1A1#4	TGCTCCAGTCAACGTTACAA
sgEEF1A1#5	AAGCCACTTACGTTAGCACT

Table S2. CRISPRi sgiRNA sequences.

sgiRNA_ID	Sequence
sgiCtrl#1	GAGTCGGGTAAATAGACAA
sgiCtrl#2	CTACGCCCGGGGGAAAAGA
sgiRUVBL1#1	GGCCAGGAGAACGCGCGAG
sgiRUVBL1#2	AACGGCCGCATGGTAACTC

Table S3. Summary of RNAi and CRISPR library screens in EwS.

Types	Scopes	Identified targets	Libraries	References
RNAi screens	Screen for kinases involved in EwS growth	STK10 and TNK2	Kinase siRNA library (572 genes)	Arora et al., 2010 [1]
	Screen for EWSR1-FLI1 regulators	HNRNPH1 and SF3B1	Genome-scale siRNA library	Grohar et al., 2016 [2]
	Screen for effectors in EwS cell survival	LRWD1	Druggable siRNA library (6781 genes)	He et al., 2016 [3]
CRISPR-Cas9 screens	Screen for genetic dependencies in TP53 wild-type EwS cells	MDM2, MDM4, USP7, and PPM1D	Genome-scale CRISPR-Cas9 library	Stolte et al., 2018 [4]
	Screen for regulators mediating EWSR1-FLI1 stability	TRIM8		Seong et al., 2021 [5]
	Screen for modulators of LSD1 inhibition in EwS	Mitochondrial complexes III and IV		Tokarsky et al., 2022 [6]

Table modified from Li et al. 2022 [7]

Table S4. RT-qPCR primers.

Primer_ID	Sequence
RUVBL1_F	AAAGAGCGAGTAGAAGCTGGA
RUVBL1_R	CCAAGTCATGCAAGGTCACATC
EEF1A1_F	GAAGCTGGTATCTCCAAGAATGG
EEF1A1_R	CGACAATTAGTTGTTTCACACCC
GAPDH_F	CCTCCTAGGCCTTTGCCTGA
GAPDH_R	CTGAGAGGCGGGAAAGTTGG

Table S5. ChIP-qPCR primers.

Primer_ID	Sequence
RUVBL1_TSS_F	GGTGACCTCCAGTTCCTAGACC
RUVBL1_TSS_R	GGTCCGGCCCGTTAAGTT
EEF1A1_TSS_F	ACTTTCCCAGTTTACCCCGC
EEF1A1_TSS_R	CCACAGTCCCCGAGAAGTTG

Supplementary References

[1] S. Arora, I. M. Gonzales, R. T. Hagelstrom, C. Beaudry, A. Choudhary, C. Sima, R. Tibes, S. Mousses, D. O. Azorsa, *Mol Cancer* **2010**, *9*, 218, https://doi.org/10.1186/1476-4598-9-218.

[2] P. J. Grohar, S. Kim, G. O. Rangel Rivera, N. Sen, S. Haddock, M. L. Harlow, N. K. Maloney, J. Zhu, M. O'Neill, T. L. Jones, K. Huppi, M. Grandin, K. Gehlhaus, C. A. Klumpp-Thomas, E. Buehler, L. J. Helman, S. E. Martin, N. J. Caplen, *Cell Rep* **2016**, *14* (3), 598, https://doi.org/10.1016/j.celrep.2015.12.063.

[3] T. He, D. Surdez, J. K. Rantala, S. Haapa-Paananen, J. Ban, M. Kauer, E. Tomazou, V. Fey, J. Alonso, H. Kovar, O. Delattre, K. Iljin, *Gene* **2017**, *596*, 137, https://doi.org/10.1016/j.gene.2016.10.021.

[4] B. Stolte, A. B. Iniguez, N. V. Dharia, A. L. Robichaud, A. S. Conway, A. M. Morgan, G. Alexe, N. J. Schauer, X. Liu, G. H. Bird, A. Tsherniak, F. Vazquez, S. J. Buhrlage, L. D. Walensky, K. Stegmaier, *J Exp Med* **2018**, *215* (8), 2137, https://doi.org/10.1084/jem.20171066.

[5] B. K. A. Seong, N. V. Dharia, S. Lin, K. A. Donovan, S. Chong, A. Robichaud, A. Conway, A. Hamze, L. Ross, G. Alexe, B. Adane, B. Nabet, F. M. Ferguson, B. Stolte, E. J. Wang, J. Sun, X. Darzacq, F. Piccioni, N. S. Gray, E. S. Fischer, K. Stegmaier, *Cancer Cell* **2021**, *39* (9), 1262, https://doi.org/10.1016/j.ccell.2021.07.003.

[6] E. J. Tokarsky, J. C. Crow, L. M. Guenther, J. Sherman, C. Taslim, G. Alexe, K. I. Pishas, G. Rask, B. S. Justis, A. Kasumova, K. Stegmaier, S. L. Lessnick, E. R. Theisen, *Mol Cancer Res* **2022**, https://doi.org/10.1158/1541-7786.MCR-22-0027.

[7] M. Li, C. W. Chen, *Biomedicines* **2022**, *10* (6), https://doi.org/10.3390/biomedicines10061325.
